# Global Trends in Deprescribing Benzodiazepine Receptor Agonists in Older Adults: A Dual-Database Bibliometric and Visual Analysis

**DOI:** 10.3390/healthcare14172856

**Published:** 2026-09-04

**Authors:** Lei Liu, Yuqiang Lu, Lei Nie, Chu Wang, Lijuan Wang, Bo Chen, Zhongwei Guo, Yan Zhang, Zhenzhong Zhang

**Affiliations:** 1 The Integrated Traditional Chinese and Western Medicine School of Clinical Medicine, Zhejiang Chinese Medical University, Hangzhou 310053, China; liulei0609h@163.com (L.L.);; 2 Tongde Hospital Affiliated to Zhejiang Chinese Medical University (Tongde Hospital of Zhejiang Province), Hangzhou 310012, China; luyuqiang@126.com (Y.L.);

**Keywords:** benzodiazepine receptor agonists, deprescribing, older adults, medication safety, potentially inappropriate medications, primary care, bibliometric analysis

## Abstract

**Highlights:**

**What are the main findings?**

- Research on benzodiazepine receptor agonist (BZRA) deprescribing among older adults showed an overall increase in publication output, with output during 2021–2025 generally higher than in preceding years; the United States, Canada, and Australia were the leading contributors.
- Bibliometric patterns indicate a thematic evolution from withdrawal and discontinuation towards broader medication safety, prescription optimization, patient education, and implementation in primary care and long-term care settings.

**What are the implications of the main findings?**

- The literature increasingly frames BZRA deprescribing as part of a broader patient-centered medication-safety and prescription-optimization process rather than as an isolated discontinuation event.
- Future research should evaluate individualized tapering approaches, nonpharmacological support, multidisciplinary care models, and standardized long-term patient-centered and safety outcomes.

**Abstract:**

**Objectives**: Long-term or inappropriate use of benzodiazepine receptor agonists (BZRAs) in older adults is a medication-safety concern. This study mapped trends, contributors, knowledge structures, and research hotspots of BZRA deprescribing using a comparative dual-database bibliometric framework. **Methods**: English-language articles and reviews from 1 January 2008 to 21 May 2026 were retrieved from the Web of Science Core Collection (WoSCC) and Scopus. Of the 377 WoSCC and 558 Scopus records entering topical relevance screening, 306 and 418, respectively, met the final eligibility criteria. Among the final datasets, 225 publications were shared, yielding 499 unique publications. The BIBLIO framework guided reporting. Analyses used R, bibliometrix, and VOSviewer, whereas CiteSpace-based analyses were restricted to WoSCC. Data for 2026 were partial; trend models used complete years through 2025. **Results**: Publication output showed an overall upward trend, with output during 2021–2025 generally higher than in earlier years. Quadratic models based on 2008–2025 data showed good fit for WoSCC (R^2^ = 0.8890) and Scopus (R^2^ = 0.9104). The United States, Canada, and Australia were leading contributors, and the University of Montreal was the leading institution (WoSCC, *n* = 19; Scopus, *n* = 18). Across both databases, themes evolved from withdrawal, discontinuation, and potentially inappropriate prescribing toward medication safety, prescription optimization, medication review, patient education, and implementation in primary care and long-term care settings. **Conclusions**: BZRA deprescribing research has broadened from drug discontinuation toward a patient-centered medication-safety and prescription-optimization framework. Future research should evaluate individualized tapering, nonpharmacological support, multidisciplinary care models, and standardized long-term patient-centered and safety outcomes.

## 1. Introduction

Population aging has intensified the clinical challenges associated with chronic multimorbidity, polypharmacy, and potentially inappropriate medication use among older adults [1]. Sedative-hypnotic medications are commonly prescribed for sleep disorders, anxiety, neuropsychiatric symptoms, and related problems in long-term care settings [1,2,3]. Among these agents, benzodiazepine receptor agonists (BZRAs), including traditional benzodiazepines and Z-drugs such as zolpidem, zopiclone, eszopiclone, and zaleplon, are widely used. Although BZRAs may provide short-term relief for difficulty initiating or maintaining sleep, age-related pharmacokinetic and pharmacodynamic changes make older adults particularly vulnerable to adverse outcomes associated with long-term or inappropriate use, including cognitive impairment, falls, delirium, and drug dependence [4,5]. Accordingly, the long-term use of BZRAs in older adults has become a major concern in geriatrics, sleep medicine, clinical pharmacy, and primary care [6,7].

In recent years, multiple guidelines and medication appropriateness criteria have emphasized the cautious use of BZRAs in older adults. The 2023 American Geriatrics Society Beers Criteria identify benzodiazepines and most Z-drugs as potentially inappropriate medications for older adults because of their associations with cognitive impairment, delirium, falls, fractures, motor vehicle crashes, and drug dependence [8]. The updated STOPP/START criteria further support the systematic identification of potentially inappropriate prescriptions in older adults and indicate that long-term sedative-hypnotic use should be evaluated according to indication, treatment duration, risk–benefit balance, and frailty status [9]. Although Z-drugs are sometimes considered alternatives to traditional benzodiazepines, systematic reviews and meta-analyses suggest that they are also associated with increased risks of falls, fractures, and related injuries in older adults [10]. The earlier psychiatric literature also highlighted a gap between education and the appropriate use of benzodiazepines, emphasizing the continuing importance of prescribing education [11]. Concerns about inappropriate BZRA use also extend to misuse and diversion, including online availability and the diversion of prescribed benzodiazepines and Z-drugs into broader psychoactive-drug markets [12,13]. Therefore, BZRA management should not be limited to symptom control for insomnia but should be considered within broader medication safety and prescription optimization.

Nonpharmacological interventions are an important component of chronic insomnia management. Guidelines from the American Academy of Sleep Medicine and other sleep medicine organizations recommend cognitive behavioral therapy for insomnia (CBT-I) as the first-line treatment for chronic insomnia [14,15]. Compared with pharmacotherapy alone, CBT-I targets sleep-related behaviors and cognitions and may provide more sustained benefits. However, the real-world implementation of CBT-I remains limited by shortages of trained professionals, restricted service accessibility, variation in patient adherence, and differences across care settings. As a result, some older adults continue to receive BZRAs over extended periods, sometimes in the context of repeated prescriptions or concomitant use with other central nervous system depressants. Reducing BZRA-related harms while maintaining adequate management of sleep and neuropsychiatric symptoms has therefore become an important issue in medication safety and rational prescribing for older adults.

Within this context, deprescribing has emerged as a key strategy for managing potentially inappropriate medication use in older adults. Deprescribing is not simply medication discontinuation. It is a planned, supervised process of dose reduction or medication cessation based on a comprehensive assessment of the indication, effectiveness, adverse effects, treatment goals, and patient preferences [16]. For BZRAs, evidence-based clinical practice guidelines recommend periodic reassessment of the need for continued use in older adults and consideration of gradual dose reduction or discontinuation when the risks outweigh the benefits [17]. Recent clinical guidance on hypnotic switching and deprescribing further emphasizes that BZRA tapering should avoid abrupt discontinuation and should be individualized according to drug type, dose, duration of use, withdrawal symptoms, and patient preferences [18,19].

Although systematic reviews, clinical guidelines, and randomized controlled trials have summarized the effectiveness and clinical management of BZRA deprescribing, they generally focus on specific interventions, drug classes, or care settings. Such evidence syntheses do not fully characterize the structural development of the research field, including its major contributors, collaboration networks, intellectual foundations, and changes in research themes over time. Bibliometric and visual analyses can complement conventional evidence synthesis by quantitatively mapping these structural and temporal patterns [20,21]. VOSviewer, CiteSpace, and the R package bibliometrix have been widely used in biomedical bibliometric studies to construct collaboration networks, analyze keyword co-occurrence and co-citation relationships, identify emerging topics, and visualize changes in research frontiers [22,23].

WoSCC and Scopus provide complementary perspectives for bibliometric analysis. WoSCC offers selective citation indexing and a well-established cited-reference structure, whereas Scopus provides broader journal, multidisciplinary, and regional coverage [24]. Comparative analysis of the two databases can therefore broaden literature coverage and reveal database-specific differences rather than relying on a single indexing system. An exploratory extension of the search period to 2000 showed that relevant publications before 2008 were sparse and intermittent, whereas a more sustained pattern of publication emerged thereafter. Accordingly, 2008 was retained as a pragmatic starting point for characterizing the subsequent development of the field. Therefore, this study used a comparative dual-database bibliometric framework, supplemented by database-specific visual analyses where appropriate, to map publication trends, major contributors, collaboration patterns, knowledge structures, and research hotspots in BZRA deprescribing among older adults. This study aims to characterize the structural and thematic evolution of the field and identify priorities for future research on medication safety, prescription optimization, and deprescribing implementation.

## 2. Materials and Methods

### 2.1. Data Sources and Search Period

This study adopted a dual-database bibliometric framework. Independent searches, screening, data export, and database-specific analyses were conducted using the Web of Science Core Collection (WoSCC) and Scopus to examine the bibliometric characteristics of research on benzodiazepine receptor agonist (BZRA) deprescribing among older adults. The final searches in both databases were completed on 21 May 2026 to minimize the influence of daily database updates on retrieval results. The search period was set from 1 January 2008 to 21 May 2026.

### 2.2. Inclusion and Exclusion Criteria

This study included English-language articles and reviews related to BZRA deprescribing among older adults. Letters, conference abstracts, conference papers, editorials, book chapters, notes, and other document types not classified as articles or reviews were excluded.

Older adults were operationally defined as individuals aged ≥65 years. Studies involving mixed-age populations were included when participants aged ≥65 years accounted for at least 50% of the study population, when data for this age group were separately extractable, or when the intervention or policy specifically targeted older adults and reported age-stratified BZRA-related outcomes. When eligibility could not be determined from the reported age information, the full text was reviewed.

BZRAs were defined as traditional benzodiazepines and Z-drugs. Eligible publications were required to address BZRA dose reduction, tapering, discontinuation, withdrawal management, or interventions and implementation strategies explicitly aimed at reducing or stopping BZRA use. Studies reporting only prescribing prevalence, medication utilization, or BZRA-related adverse outcomes without a deprescribing component were excluded. Studies addressing multiple medication classes were retained only when BZRAs constituted an identifiable deprescribing target.

### 2.3. Search Strategy and Screening

The search strategy combined three principal concepts: (1) older adults, (2) BZRAs and related hypnotics, and (3) deprescribing or medication withdrawal. Within each concept block, synonyms, spelling variants, and related terms were combined using the Boolean operator OR, and the three concept blocks were subsequently combined using AND. In WoSCC, the search was conducted using the Topic Search (TS) field, whereas in Scopus the TITLE-ABS-KEY field was used. Where applicable, proximity operators were used to link discontinuation-, reduction-, tapering-, or withdrawal-related terms with BZRA-related terms (NEAR/5 in WoSCC and W/5 in Scopus). The complete database-specific search strings, including all individual terms, Boolean and proximity operators, and field tags, are provided in Supplementary Table S1.

Two researchers independently performed literature searches and eligibility screening, and disagreements were resolved through discussion and consensus. Following database-level restrictions on publication period, document type, and language, all remaining records underwent title- and abstract-level screening for topical relevance according to the predefined population, medication, and deprescribing criteria. Full texts were reviewed when eligibility could not be determined from the title and abstract alone.

In WoSCC, the search initially identified 438 records. Restriction to articles and reviews yielded 392 records, and further restriction to English-language publications resulted in 377 records entering topical relevance screening. Of these, 71 were excluded after thematic assessment, leaving 306 publications for the final WoSCC analysis. WoSCC records were exported in plain-text (.txt) format as full records with cited references.

In Scopus, the initial search identified 803 records. Restriction to the study period yielded 638 records, restriction to articles and reviews yielded 589 records, and restriction to English-language publications resulted in 558 records entering topical relevance screening. Of these, 140 were excluded after thematic assessment, leaving 418 publications for the final Scopus analysis. Scopus records were exported in comma-separated values (.csv) format with complete bibliographic information and cited references.

Cross-database overlap was assessed using the final database-specific datasets. Records were matched primarily by DOI; records without a matching DOI were additionally compared using normalized title, first author, and publication year. Overlapping publications were quantified but retained within their respective WoSCC and Scopus datasets because the two databases were analyzed independently.

**Figure 1:**
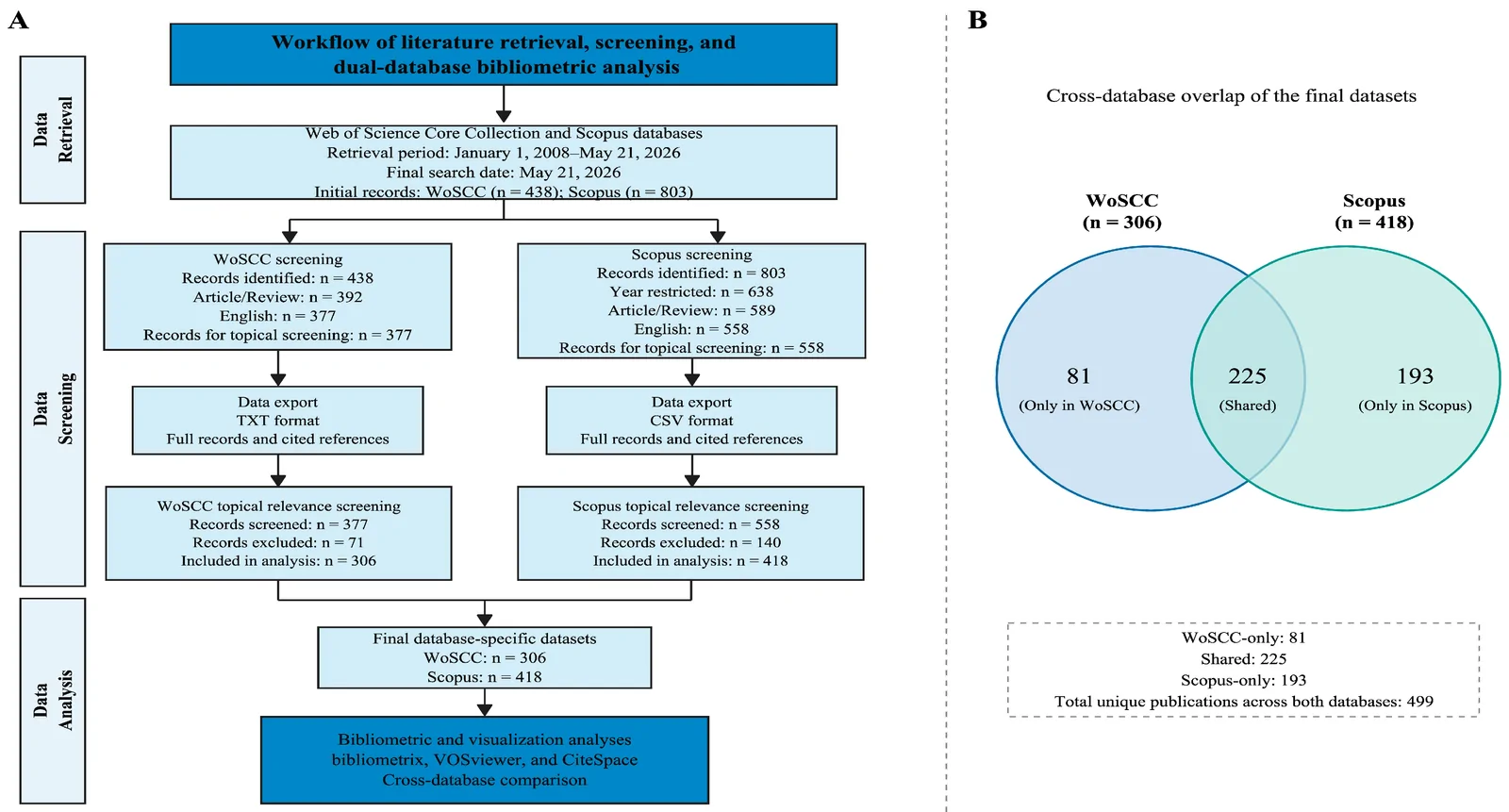
Workflow of literature retrieval, screening, and database-specific bibliometric analysis. Panel (**A**) presents the screening and analysis workflows for WoSCC and Scopus. Panel (**B**) shows cross-database overlap in the final datasets: 225 shared publications (73.5% of WoSCC and 53.8% of Scopus), 81 publications unique to WoSCC (26.5%), and 193 unique to Scopus (46.2%).

The literature retrieval, screening process, and cross-database overlap are summarized in Figure 1. Reporting of the bibliometric review was guided by the BIBLIO framework for biomedical bibliometric reviews [20], and the corresponding checklist is provided in Supplementary Table S3.

### 2.4. Data Analysis

Bibliometric analyses were conducted separately for the WoSCC and Scopus datasets using VOSviewer (version 1.6.17), CiteSpace (version 6.4.R1), R software (version 4.5.2), and the R package bibliometrix (version 3.2.1).

Keyword analyses were based on the Author Keywords field (DE) in WoSCC and the Author Keywords field in Scopus. Before visualization and trend analyses, institutional names, country/region names, and author keywords were harmonized using predefined thesaurus files to reduce the influence of spelling variants, abbreviations, and synonymous terms. For example, institutional naming variants were standardized across databases; “BZD” and “BZDs” were harmonized as “benzodiazepines”; and “deprescription” and “de-prescribing” were harmonized as “deprescribing”. The same standardization rules were applied to both databases. Detailed standardization and thesaurus rules, together with excluded non-informative terms, are provided in Supplementary Table S2.

VOSviewer was used to construct collaboration networks of countries/regions, institutions, and authors; co-cited author networks; and keyword co-occurrence networks [25]. The minimum threshold was set at three publications for country/region and author collaboration networks, five publications for institutional collaboration networks, five occurrences for keyword co-occurrence networks, and 20 co-citations for co-cited author networks. To assess the robustness of the network structures to threshold selection, sensitivity analyses were performed using lower and higher inclusion thresholds around the primary settings. Changes in node retention, network connectivity, and the overall clustering patterns were examined across parameter settings. The major contributors, principal connections, and overall network structures remained broadly stable, although lower thresholds retained additional low-frequency peripheral nodes. The primary thresholds were therefore retained to balance network coverage and visual interpretability. The same threshold criteria were applied to WoSCC and Scopus. Fractional counting was used for network construction, with association-strength normalization and a clustering resolution of 1.00. In the network maps, node size represents publication, occurrence, or co-citation frequency, whereas links and link thickness indicate relationships and their strengths.

For country-level analyses, the corresponding-author country was used to characterize publication output and international collaboration. To account for contributions from multinational publications, an additional analysis based on all author affiliations was conducted using both full and fractional counting. Under fractional counting, a publication involving k countries/regions contributed 1/k to each participating country/region.

CiteSpace was used to analyze reference co-citation networks, citation bursts, keyword clusters, and dual-map overlays of journals [26,27]. CiteSpace analyses were conducted using the WoSCC dataset to maintain a consistent cited-reference structure across these analyses. For temporal analyses, the time span was set from 2008 to 2025, with one year per slice, excluding the incomplete 2026 observation period. Nodes were selected using the g-index (k = 25), and Pathfinder pruning was applied to both the sliced networks and the merged network. Keyword clusters were labeled using the log-likelihood ratio (LLR) method, and cluster validity was evaluated using modularity Q and the weighted mean silhouette S (Q = 0.6762; S = 0.861). The minimum duration for citation bursts was set to three years.

The R package bibliometrix was used for descriptive bibliometric analysis and thematic trend visualization [28]. Analyses included annual publication output, country/region distribution, institutional distribution, author productivity, source journals, highly cited publications, and keyword frequency. Annual publication trends were fitted using quadratic polynomial regression in R, and the coefficient of determination (R^2^) was used to assess goodness of fit. To assess the potential influence of the incomplete 2026 observation period, a sensitivity analysis was performed by refitting the annual publication trend models using complete calendar years from 2008 to 2025. The partial 2026 data were retained for descriptive presentation but excluded from regression fitting.

Source-journal analyses included publication counts, total citations, and citations per document. For highly cited publications, citations per year were additionally reported to account for differences in publication age. Citation counts were based on the values reported by each database, and self-citations were not excluded. Keyword trending-topic analyses were based on the harmonized author–keyword datasets and complete calendar years through 2025. Journal Impact Factor data were obtained from the 2025 Journal Citation Reports and were used as a journal-level contextual indicator.

## 3. Results

### 3.1. Overview of Publications on BZRA Deprescribing Among Older Adults

**Figure 2:**
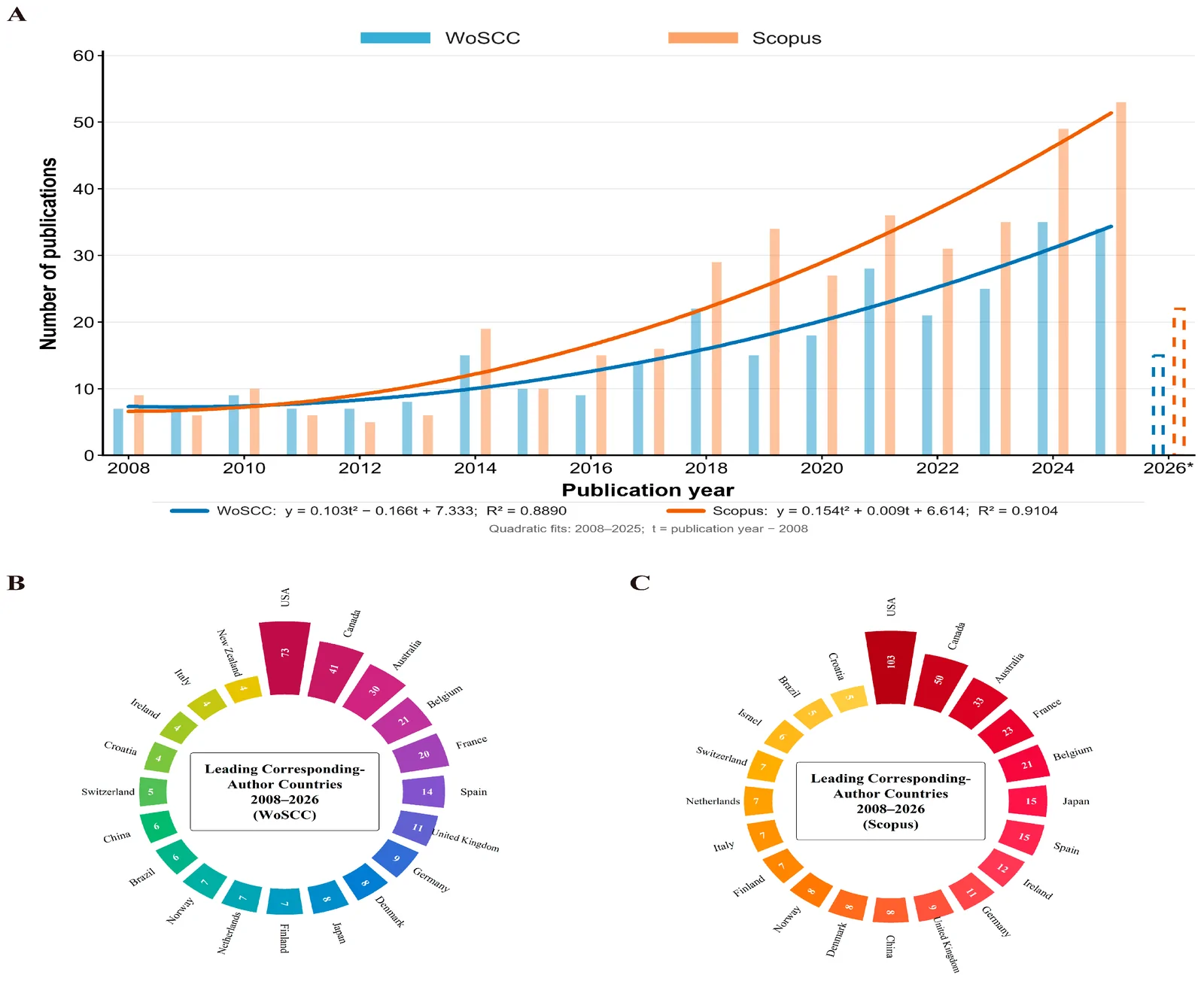
Annual publication trends and corresponding-author country distributions in WoSCC and Scopus. (**A**) Overlaid annual publication trajectories in WoSCC and Scopus, with quadratic regression fitted using complete calendar years from 2008 to 2025. The 2026 publication counts represent records available up to 21 May 2026 and were excluded from regression fitting. The solid curves represent the quadratic regression fits for 2008–2025, whereas the dashed outlines indicate the partial publication counts for 2026. The asterisk (*) indicates that 2026 represents a partial year. (**B**) Distribution of the leading corresponding-author countries in WoSCC. (**C**) Distribution of the leading corresponding-author countries in Scopus.

A total of 306 and 418 eligible publications were included from WoSCC and Scopus, respectively. Across the complete calendar years from 2008 to 2025, the annual number of publications on BZRA deprescribing among older adults showed an overall fluctuating upward trend in both databases (Figure 2A). In WoSCC, annual output remained relatively low between 2008 and 2013, ranging from 7 to 9 publications per year. The number increased to 15 in 2014 and subsequently fluctuated at a higher level, reaching a peak of 35 publications in 2024 and remaining high at 34 publications in 2025. A similar pattern was observed in Scopus, where annual output ranged from 5 to 10 publications between 2008 and 2013, increased to 19 in 2014, and reached a peak of 53 publications in 2025. Publication output during 2021–2025 was generally higher than that in the preceding years in both databases. Quadratic polynomial regression based on complete calendar years from 2008 to 2025 showed good model fit for both WoSCC (R^2^ = 0.8890) and Scopus (R^2^ = 0.9104), indicating that the fitted curves adequately captured the observed publication trajectories. Because the 2026 data were available only up to the search date, they were excluded from the regression analysis and are presented separately in Figure 2A; therefore, the 2026 publication counts should not be directly compared with those for complete calendar years.

Regarding the distribution of countries among corresponding authors, the United States ranked first in both databases and was the leading contributor in this field, with 73 publications in WoSCC and 103 in Scopus. In WoSCC, the next most productive countries were Canada (*n* = 41), Australia (*n* = 30), Belgium (*n* = 21), and France (*n* = 20). In Scopus, the leading countries after the United States were Canada (*n* = 50), Australia (*n* = 33), France (*n* = 23), and Belgium (*n* = 21) (Table 1A; Figure 2B,C). Although the United States had the highest publication output, its proportion of multiple-country publications (MCP%) was 12.3% in WoSCC and 13.6% in Scopus. By contrast, Canada had MCP% values of 17.1% and 24.0%, respectively, while Australia had values of 26.7% and 21.2%, indicating relatively active participation in international collaboration among publications led by these countries. Belgium also showed comparatively high MCP proportions, particularly in Scopus, where 47.6% of its corresponding-author publications involved international collaboration.

To provide a broader view of country contributions, an additional analysis based on all author affiliations was performed. Under full counting, the United States, Canada, and Australia were the three leading contributors in both databases. After fractional allocation of publications according to the number of countries represented in each paper, these countries remained the top three contributors, with fractional publication counts of 75.22, 42.94, and 27.33 in WoSCC and 111.00, 56.86, and 37.29 in Scopus, respectively (Table 1B). The consistency of the leading countries across corresponding-author, full-count, and fractional-count analyses further indicates the prominent contributions of the United States, Canada, and Australia to BZRA deprescribing research.

**Figure 3:**
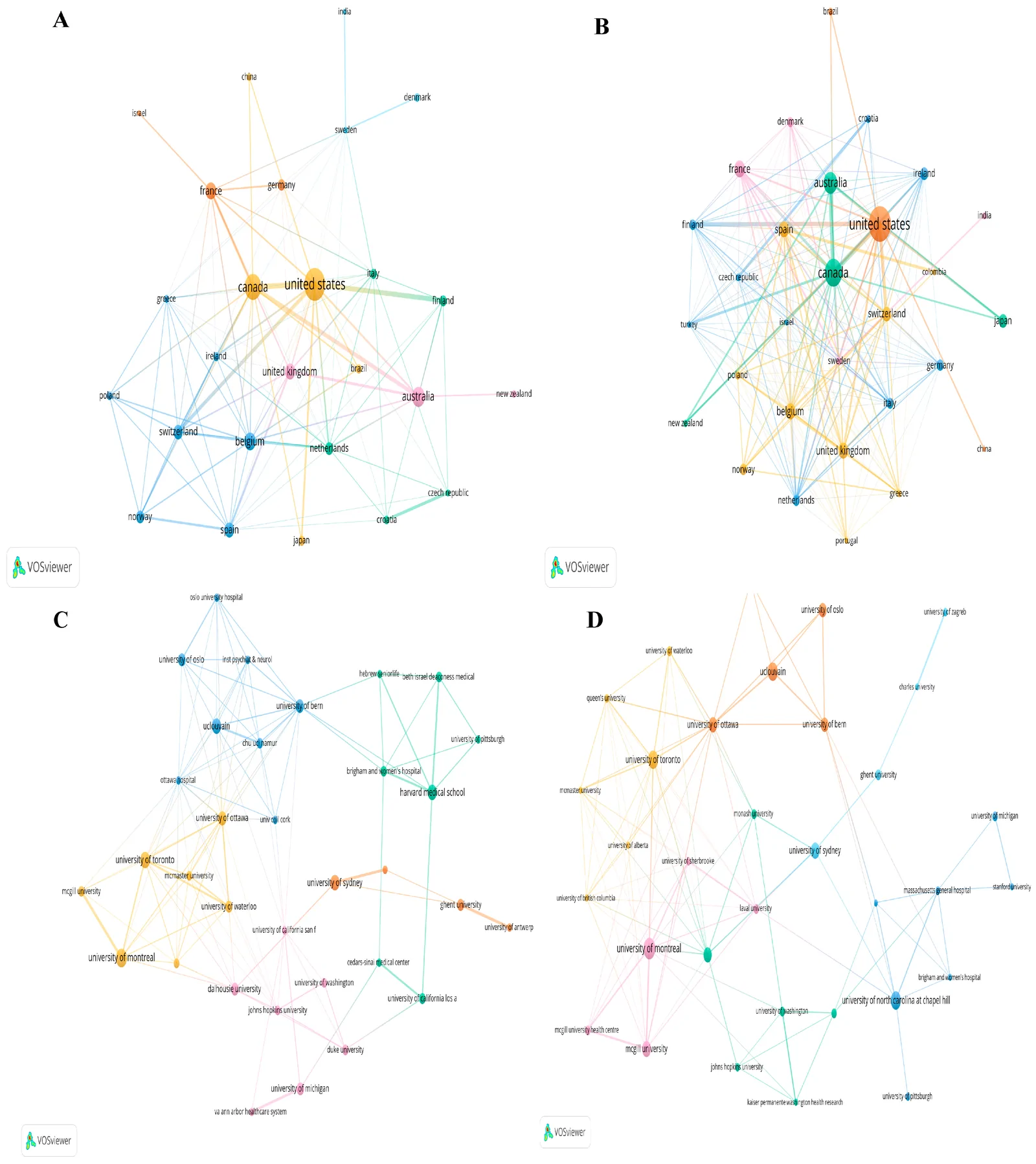
Country/region and institutional collaboration networks in the Web of Science Core Collection (WoSCC) and Scopus, generated using VOSviewer. (**A**) Country/region collaboration network in WoSCC. (**B**) Country/region collaboration network in Scopus. (**C**) Institutional collaboration network in WoSCC. (**D**) Institutional collaboration network in Scopus. Node size represents publication output, links indicate collaboration relationships, link thickness reflects collaboration strength, and colors distinguish network clusters.

Country collaboration network analysis further showed that the United States, Canada, and Australia were the main collaboration nodes in both databases (Figure 3A, B). These countries were connected with multiple research partners and occupied relatively central positions in the collaboration networks, indicating that international collaboration in this field was largely concentrated around several high-output countries. Nevertheless, collaborative links also extended across Europe, Asia, and other regions, indicating broad international participation in this field.

At the institutional level, the leading research institutions were mainly located in Canada, the United States, Australia, and Europe. In WoSCC, the University of Montreal had the highest publication output (*n* = 19), followed by the University of Toronto (*n* = 15), Harvard Medical School (*n* = 14), UCLouvain (*n* = 14), and the University of Sydney (*n* = 14). In Scopus, the University of Montreal ranked first (*n* = 18), followed by the University of Toronto (*n* = 15), UCLouvain (*n* = 15), and the University of North Carolina at Chapel Hill (*n* = 15) (Table 2). Institutional collaboration networks further showed that Canadian universities occupied important positions in both databases (Figure 3C,D). In WoSCC, the University of Ottawa showed particularly extensive institutional connections (degree = 16; weighted degree = 12), indicating a broad range of collaborative relationships despite not having the highest publication output. In Scopus, the University of Montreal combined the highest publication output with strong collaboration connectivity (degree = 13; weighted degree = 15). These findings indicate that institutional productivity and collaboration connectivity were related but did not necessarily coincide. Overall, Canadian universities demonstrated strong performance in BZRA deprescribing research, with both high publication productivity and central positions in institutional collaboration networks.

### 3.2. Author Contributions and Collaborative Network Patterns

Author-level analysis showed broadly consistent patterns of prolific authors across the WoSCC and Scopus datasets. In WoSCC, Spinewine A had the highest number of publications (*n* = 16), followed by Tannenbaum C (*n* = 12) and Henrard S (*n* = 10). Martin P, Kivelä SL, Vahlberg T, and Evrard P each contributed seven publications. In terms of total citations, Tannenbaum C ranked first (citations = 873), despite ranking second in publication output, followed by Martin P (citations = 753). In Scopus, Spinewine A also ranked first in publication output (*n* = 16), followed by Tannenbaum C and Niznik JD (*n* = 11 each), Henrard S (*n* = 10), and Turner JP (*n* = 8). Tannenbaum C had the highest citation count in Scopus (citations = 1159), followed by Tamblyn R (citations = 1034) and Hilmer SN (citations = 339) (Table 3). Overall, Spinewine A, Tannenbaum C, and Henrard S were among the most consistently productive authors across both databases, while Tannenbaum C showed substantial citation impact in both datasets. Citation counts differed between WoSCC and Scopus and should therefore be interpreted as database-specific measures rather than as directly comparable estimates of author impact.

**Figure 4:**
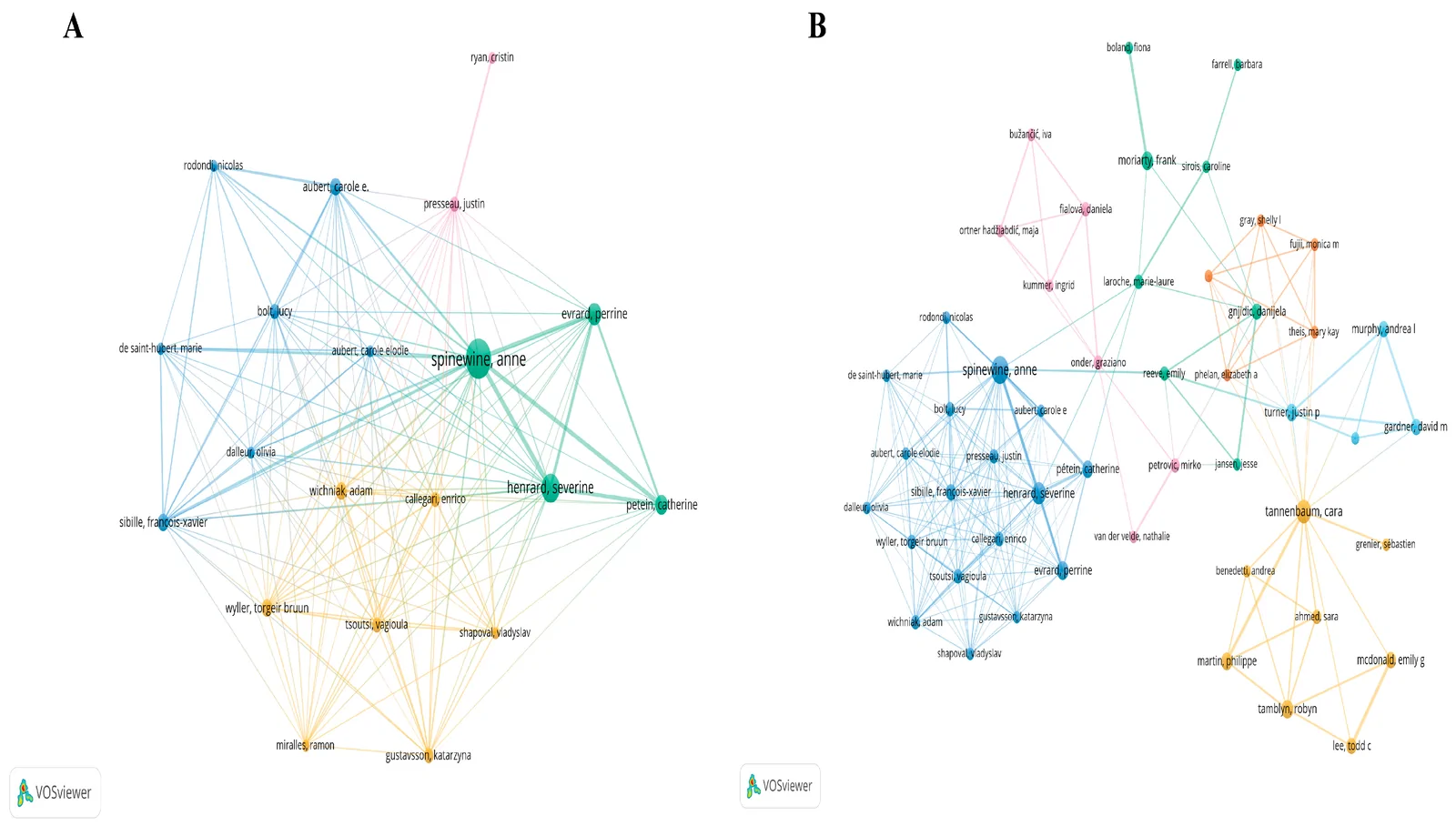
Author collaboration networks in the Web of Science Core Collection (WoSCC) and Scopus, generated using VOSviewer. (**A**) WoSCC. (**B**) Scopus. Node size represents author publication output, links indicate co-authorship relationships, link thickness reflects collaboration strength, and colors distinguish author collaboration clusters.

The author collaboration networks revealed a multi-cluster structure rather than a centralized network dominated by a single author or research team. In the WoSCC author collaboration network, Spinewine A and Henrard S occupied prominent positions within a relatively dense collaborative core, together with authors such as Pétein C, Evrard P, and Aubert CE. Collaboration within individual author groups appeared relatively dense, whereas links between different groups were more limited (Figure 4A). In the Scopus author collaboration network, the collaboration clusters appeared more dispersed. Spinewine A and Henrard S remained prominent within one major collaborative group, while Tannenbaum C was located within another distinct cluster together with authors including Martin P, Tamblyn R, and several other collaborators (Figure 4B). Overall, the two databases showed a collaboration pattern characterized by several relatively cohesive research teams, with stronger within-group connections than between-group links, suggesting that cross-team collaboration in BZRA deprescribing research remains comparatively limited.

### 3.3. Source Journals and Citation Pathways

To evaluate the publication performance and citation impact of source journals in this field, Bibliometrix was used to analyze journal publication output, total citations (TC), and citations per document (CPD), and ggplot2 (version 4.0.1) was used to generate bubble plots for visualization. High-output journals serve as the main publication venues for research on BZRA deprescribing among older adults, whereas highly cited journals are key venues for disseminating influential evidence.

**Figure 5:**
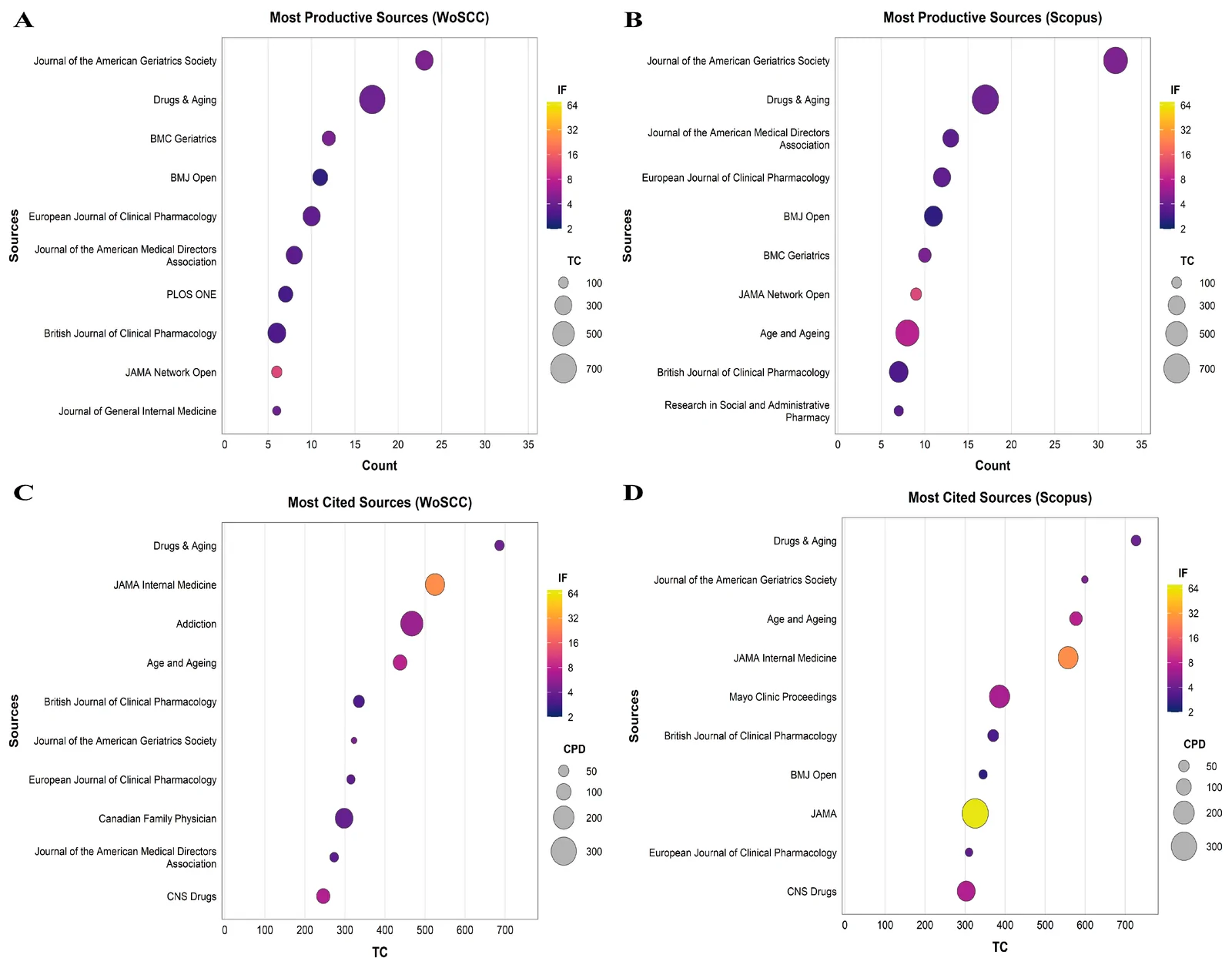
Publication output and citation performance of leading source journals in WoSCC and Scopus. (**A**) Most productive sources in WoSCC. (**B**) Most productive sources in Scopus. (**C**) Most cited sources in WoSCC. (**D**) Most cited sources in Scopus. Bubble size represents total citations in (**A**,**B**) and citations per document in panels (**C**,**D**); color represents the 2025 Journal Impact Factor as a journal-level contextual indicator.

In WoSCC, the 306 eligible publications were published across 130 academic journals. The journal with the highest number of publications was the *Journal of the American Geriatrics Society* (*n* = 23), followed by *Drugs & Aging* (*n* = 17) and *BMC Geriatrics* (*n* = 12). In Scopus, the 418 eligible publications were published across 208 academic journals. The *Journal of the American Geriatrics Society* also ranked first (*n* = 32), followed by *Drugs & Aging* (*n* = 17) and the *Journal of the American Medical Directors Association* (*n* = 13) (Table 4; Figure 5A,B). Results from both databases indicate that the *Journal of the American Geriatrics Society* and *Drugs & Aging* were the most prominent high-output journals in this field. These high-output journals were mainly distributed across geriatrics, clinical pharmacology, drug safety, and related interdisciplinary medical fields.

In terms of total citations, *Drugs & Aging* ranked first in both WoSCC (TC = 686) and Scopus (TC = 727). In WoSCC, it was followed by *JAMA Internal Medicine* (TC = 525) and *Addiction* (TC = 467), whereas in Scopus, the next most cited journals were the *Journal of the American Geriatrics Society* (TC = 599), *Age and Ageing* (TC = 577), and *JAMA Internal Medicine* (TC = 557) (Table 5; Figure 5C,D). CPD further distinguished journals with high cumulative citation counts from those with high citation averages per included publication. Although *Drugs & Aging* and the *Journal of the American Geriatrics Society* ranked among the leading journals by total citations, their high cumulative citation counts were partly associated with their relatively larger publication volumes. In contrast, journals with fewer included publications, such as *Addiction* in WoSCC (CPD = 233.50), *JAMA Internal Medicine* in WoSCC and Scopus (CPD = 175.00 and 185.67, respectively), and *JAMA* in Scopus (CPD = 325.00), showed high CPD values despite their smaller publication output. Taken together, the publication-output and citation-performance results indicate that the source-journal landscape included both field-specific journals with sustained publication and citation accumulation and broader medical journals that contributed fewer but highly cited papers.

**Figure 6:**
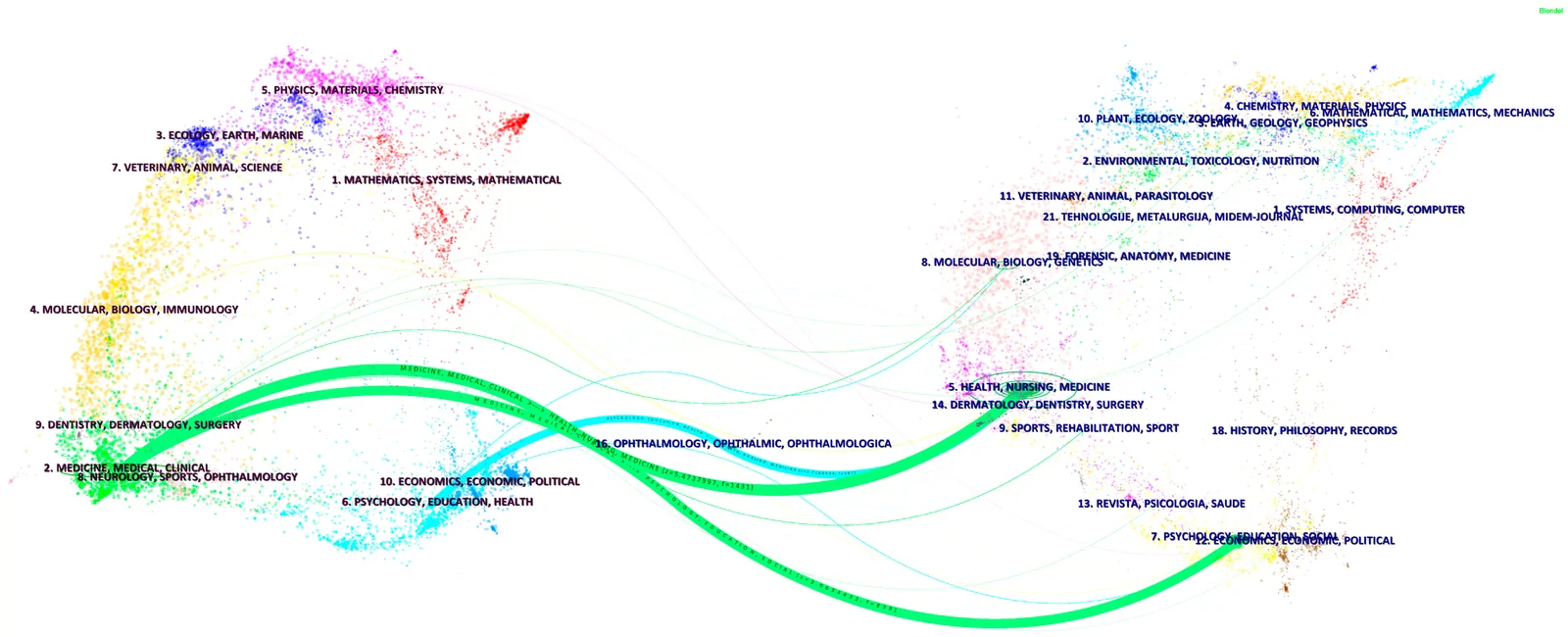
Dual-map overlay of the citation landscape based on WoSCC; citing journal clusters are shown on the left, cited journal clusters on the right, and colored lines represent citation paths between disciplines.

The WoSCC-based dual-map overlay of journals further illustrated the disciplinary distribution of citing and cited journals (Figure 6). The citing journals on the left were mainly located in the “Medicine, Medical, Clinical” and “Psychology, Education, Health” domains, whereas the cited journals on the right were concentrated in the “Health, Nursing, Medicine” and “Psychology, Education, Social” domains. The main citation pathways extended from clinical medicine and health-related journals to journals in nursing and health sciences, psychology and behavioral sciences, and social medicine. These findings suggest that research on BZRA deprescribing among older adults is primarily published in clinical medicine and health science journals, while its knowledge base draws on multidisciplinary evidence from nursing, psychology, behavioral science, and social medicine.

### 3.4. Highly Cited Publications, Co-Citation Networks, and Evolution of the Knowledge Base

Highly cited publications indicate the core evidence base and influential studies in the field. The top 10 most-cited publications in WoSCC each received at least 134 citations, whereas the top 10 most-cited publications in Scopus each received at least 157 citations (Table 6 and Table 7). The full rankings of the top 20 most-cited publications, including DOI information and annualized citation rates, are provided in Supplementary Tables S4 and S5. Seven publications appeared among the top 10 in both databases, indicating substantial consistency in the core highly cited literature. The most frequently cited publication in both databases was the EMPOWER cluster randomized trial by Tannenbaum et al., published in *JAMA Internal Medicine* (WoSCC: TC = 495; Scopus: TC = 531). This study evaluated direct patient education as a strategy to promote benzodiazepine discontinuation among older adults [29].

In terms of thematic focus, the highly cited literature mainly addressed the risks of long-term benzodiazepine use, medication withdrawal and discontinuation, medication safety in older adults, and structured deprescribing interventions. Lader’s studies on long-term benzodiazepine use and withdrawal management [30,31,32], together with the systematic review by Iyer et al. on medication withdrawal among adults aged ≥ 65 years (WoSCC: TC = 269; Scopus: TC = 319) [33], formed an important early evidence base for risk recognition and discontinuation. Clegg et al.’s systematic review on medications associated with delirium risk also ranked prominently in both databases (WoSCC: TC = 382; Scopus: TC = 480), linking benzodiazepine use with broader concerns regarding medication safety in older adults [34]. Reeve et al.’s systematic review further summarized evidence on interventions to deprescribe benzodiazepines and other hypnotics [35], while Pottie et al.’s evidence-based clinical practice guideline [16] and Martin et al.’s pharmacist-led D-PRESCRIBE trial [36] represented important practice-oriented developments in BZRA deprescribing. These findings indicate that the highly cited literature spans not only BZRA-related risks and withdrawal but also medication-safety assessment, intervention strategies, and clinical guidance for deprescribing.

Citation rates adjusted for publication age provided an additional perspective on citation impact. The EMPOWER trial remained the leading publication by annualized citations in both WoSCC (38.08 citations/year) and Scopus (40.85 citations/year). More recent publications also showed relatively high annual citation rates, including Martin et al.’s 2018 D-PRESCRIBE trial in Scopus (36.11 citations/year) and Pottie et al.’s 2018 guideline in WoSCC and Scopus (25.44 and 28.89 citations/year, respectively) [16,36]. Thus, although earlier publications had more time to accumulate citations, recent intervention studies and clinical guidance also attracted substantial citation attention within a shorter publication period.

Despite the substantial overlap, the database-specific publications showed some differences in thematic emphasis. WoSCC included more broadly oriented studies on deprescribing and polypharmacy, whereas Scopus placed relatively greater emphasis on benzodiazepine management and intervention strategies. Overall, both databases identified a largely shared core evidence base while providing complementary perspectives on the field.

**Figure 7:**
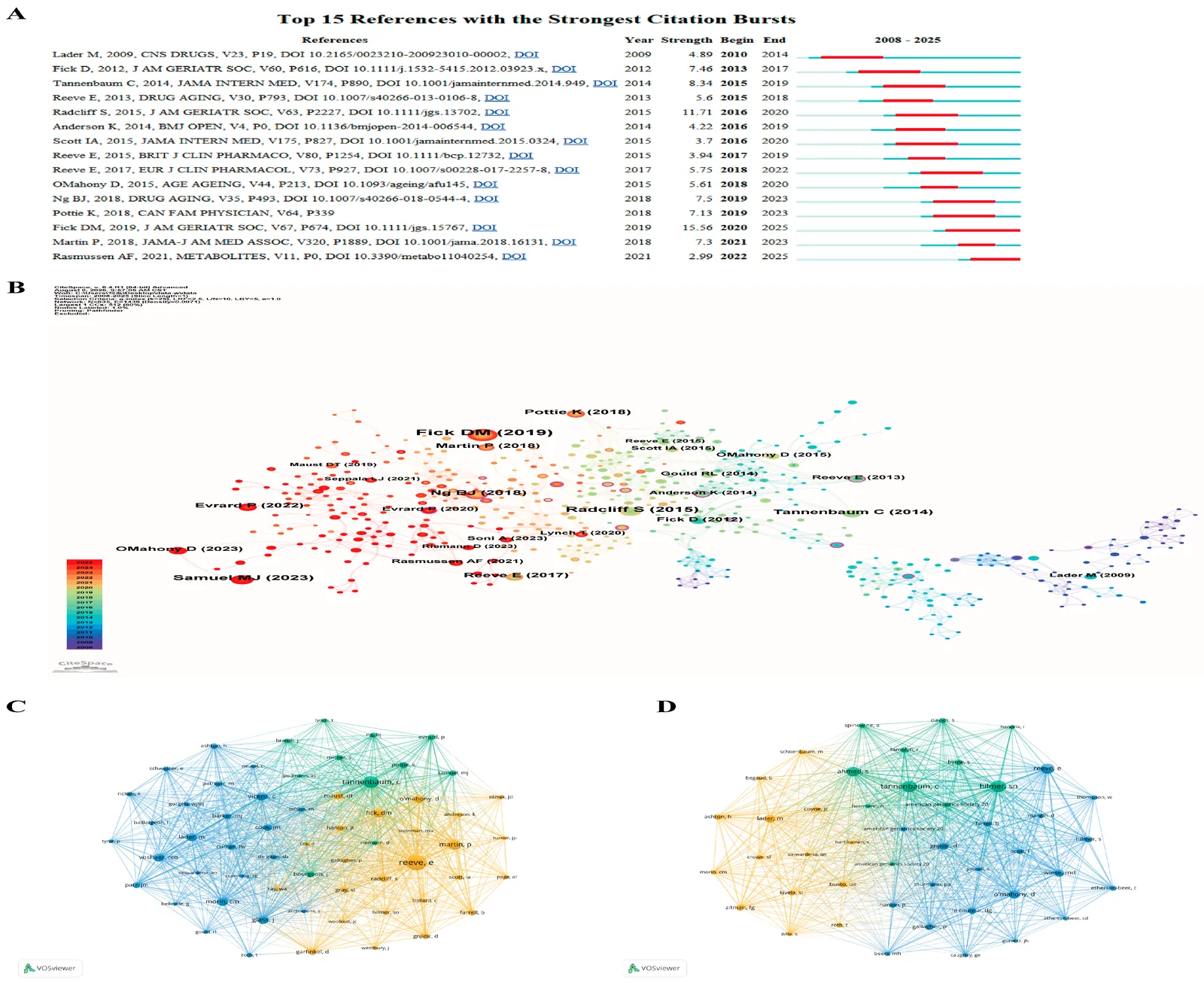
Citation bursts and co-citation networks of references and authors. (**A**) Top 15 references with the strongest citation bursts in WoSCC [16,29,30,35,36,37,38,39,40,41,42,43,44,45,46]. (**B**) Reference co-citation network in WoSCC. (**C**) Author co-citation network in WoSCC. (**D**) Author co-citation network in Scopus. CiteSpace-based temporal analyses, including citation-burst detection, were restricted to 2008–2025; the incomplete 2026 observation period was excluded.

The WoSCC-based reference co-citation network and citation-burst analysis further revealed the evolution of the knowledge base from benzodiazepine withdrawal and risk recognition to medication-safety standards for older adults and subsequently to evidence-based deprescribing strategies (Figure 7A,B). Lader’s 2009 [30] study on benzodiazepine withdrawal showed an early citation burst (burst strength = 4.89, 2010–2014), followed by the 2012 AGS Beers Criteria (burst strength = 7.46, 2013–2017). The EMPOWER study (burst strength = 8.34, 2015–2019) and the 2015 AGS Beers Criteria (burst strength = 11.71, 2016–2020) reflected growing attention to patient-centered deprescribing and medication appropriateness. More recently, the 2019 AGS Beers Criteria update showed the strongest citation burst (burst strength = 15.56, 2020–2025), while Pottie et al.’s deprescribing guideline (burst strength = 7.13, 2019–2023) and Martin et al.’s D-PRESCRIBE trial (burst strength = 7.30, 2021–2023) also showed later citation bursts. These patterns indicate that the knowledge base has progressively expanded from withdrawal management and risk identification toward medication-safety standards and structured deprescribing interventions.

Unlike prolific authors, who mainly reflect research productivity, co-cited authors help identify scholars whose work constitutes the intellectual foundation of the field. In WoSCC, Reeve E had the highest co-citation frequency (*n* = 167), followed by Tannenbaum C (*n* = 113), Martin P (*n* = 88), and Fick DM (*n* = 73). In Scopus, Hilmer SN ranked first (*n* = 104), followed by Tannenbaum C (*n* = 101), Reeve E (*n* = 86), and Ahmed S (*n* = 79) (Table 3). The co-cited author networks further showed that Reeve E and Tannenbaum C occupied prominent positions across both databases, while Martin P and Fick DM were more prominent in WoSCC and Hilmer SN in Scopus (Figure 7C,D). The prominence of Reeve E and Tannenbaum C is consistent with their influential work on deprescribing interventions and patient education. At the same time, Martin P, Fick DM, and Hilmer SN further reflect the importance of intervention research, medication-appropriateness criteria, and geriatric medication safety in the knowledge base of this field.

### 3.5. Keyword Co-Occurrence, Topic Clustering, and the Evolution of Research Frontiers

Keyword co-occurrence analysis was used to identify the main research hotspots and thematic structures in studies on BZRA deprescribing among older adults. After keyword standardization, 539 unique keywords were identified in WoSCC and 720 in Scopus. The 20 most frequent keywords in each database are listed in Table 8.

**Figure 8:**
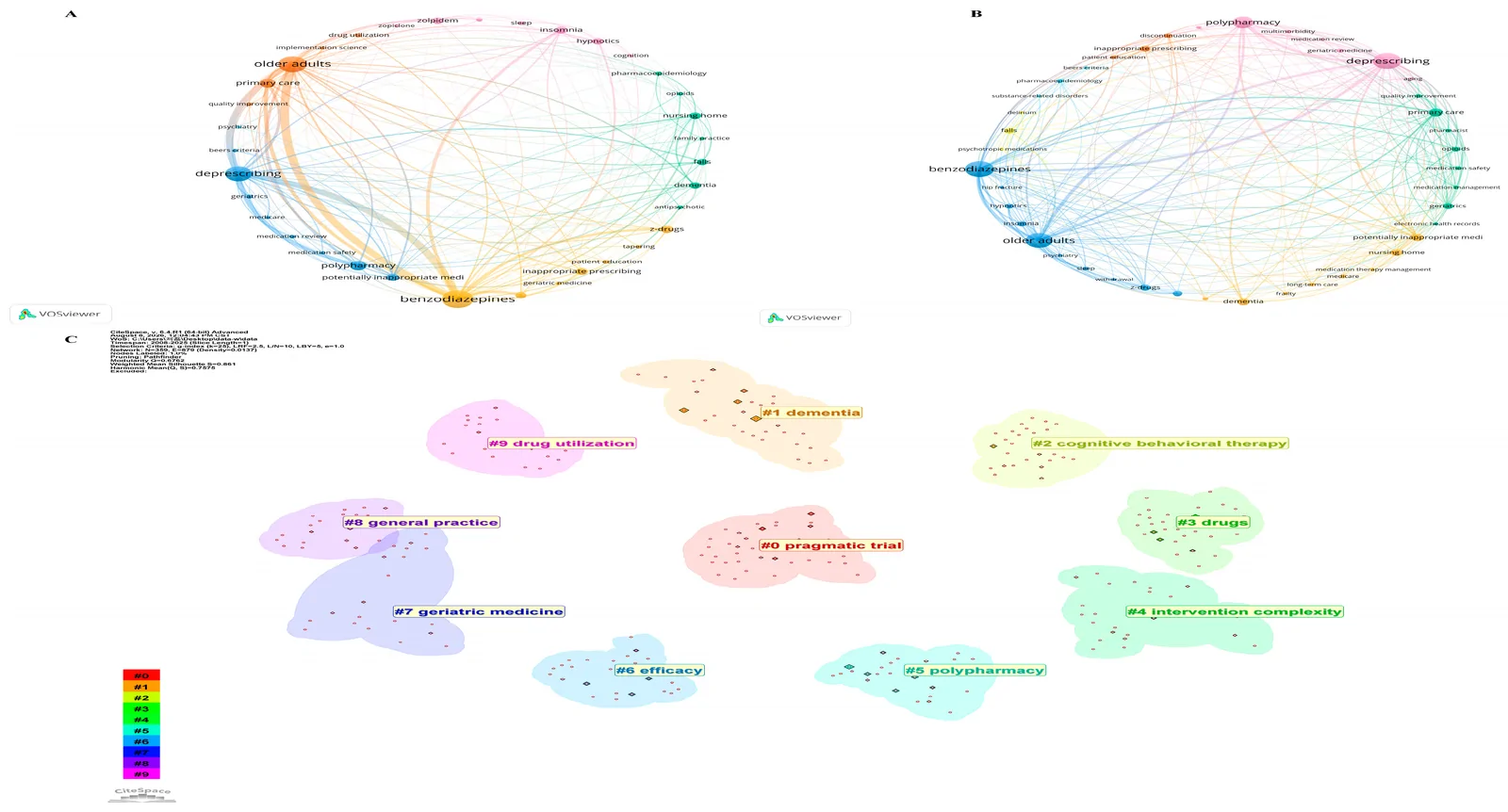
Keyword co-occurrence networks and keyword clustering map. (**A**) Keyword co-occurrence network in the Web of Science Core Collection (WoSCC), generated using VOSviewer. (**B**) Keyword co-occurrence network in Scopus, generated using VOSviewer. In panels A and B, node size represents keyword occurrence frequency, links indicate co-occurrence relationships, link thickness reflects co-occurrence strength, and colors distinguish keyword clusters. (**C**) CiteSpace keyword clustering map based on WoSCC, in which colors distinguish keyword clusters and cluster labels summarize the major thematic groups.

By integrating high-frequency keywords with keyword co-occurrence networks, several major research hotspots were identified (Figure 8A,B). First, a major theme concerned the use and discontinuation management of BZRAs and hypnotics. Representative keywords included “benzodiazepines,” “hypnotics,” “Z-drugs,” “zolpidem,” “discontinuation,” “withdrawal,” and “deprescribing.” Second, medication safety and prescription optimization in older adults constituted another important theme. Related keywords included “polypharmacy,” “potentially inappropriate medications,” “inappropriate prescribing,” and “pharmacoepidemiology,” indicating that BZRA deprescribing is closely linked with broader medication management and prescribing optimization. Third, primary care and institutional care settings represented important contexts for deprescribing research, as reflected by terms such as “primary care,” “nursing home,” and “geriatric medicine.” Fourth, insomnia management and safety-related outcomes were also prominent, with terms such as “insomnia,” “sleep,” “falls,” and “dementia.” Overall, these patterns indicate that research in this field extends beyond drug-specific discontinuation to broader issues involving medication optimization, clinical implementation, and geriatric safety.

The WoSCC-based CiteSpace clustering analysis further supported this thematic structure (Figure 8C). Clusters #0 “pragmatic trial,” #4 “intervention complexity,” and #6 “efficacy” reflected the evaluation and implementation of deprescribing interventions, while cluster #2 “cognitive behavioral therapy” represented nonpharmacological management. Other clusters, including #5 “polypharmacy,” #7 “geriatric medicine,” #8 “general practice,” and #9 “drug utilization,” linked deprescribing with broader medication management and clinical care settings. Together with clusters #1 “dementia” and #3 “drugs,” these findings show that the knowledge structure spans intervention evaluation, implementation complexity, nonpharmacological management, medication optimization, and geriatric safety.

**Figure 9:**
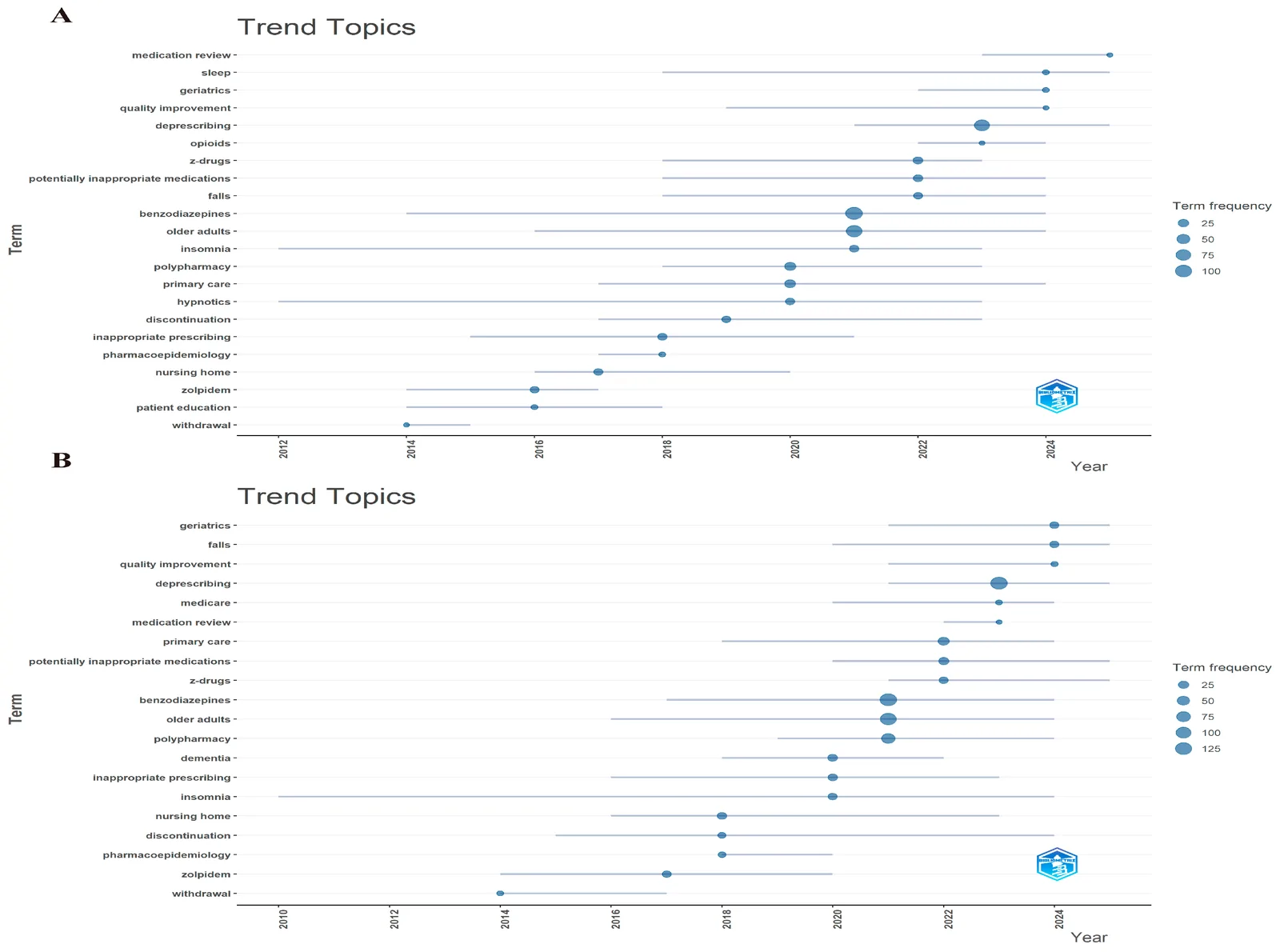
Trend topics in WoSCC and Scopus based on complete calendar years through 2025. (**A**) Trend topics in WoSCC. (**B**) Trend topics in Scopus.

Trending-topic analysis further illustrated the temporal evolution of research frontiers in this field (Figure 9A,B). Earlier research was characterized mainly by terms related to withdrawal, zolpidem, nursing-home care, and primary care. Subsequently, discontinuation, inappropriate prescribing, insomnia, and polypharmacy became more prominent. More recent topics included “deprescribing,” “medication review,” “quality improvement,” and “geriatrics,” together with safety- and medication-management-related terms such as “falls,” “sleep,” and “opioids.” Although some differences in timing and emphasis were observed between WoSCC and Scopus, both databases showed a broadly similar evolution from withdrawal and discontinuation toward medication optimization and deprescribing implementation.

Overall, the co-occurrence, clustering, and trend analyses were complementary and revealed a chronological evolution from an early focus on withdrawal and discontinuation to a broader emphasis on potentially inappropriate prescribing, medication review, safety outcomes, and the implementation of deprescribing within geriatric care.

## 4. Discussion

### 4.1. Principal Findings

This dual-database bibliometric analysis showed an overall upward trend in BZRA deprescribing research among older adults, with publication output during 2021–2025 generally higher than in the preceding years. Across WoSCC and Scopus, three patterns were consistent: publication output increased overall; research activity was mainly concentrated in the United States, Canada, Australia, and several European countries; and research themes shifted from withdrawal and discontinuation toward medication safety, prescription optimization, and healthcare implementation. These patterns may partly reflect the development of geriatric pharmacotherapy, clinical pharmacy services, deprescribing research networks, and medication-review initiatives in several of these leading countries. The comparatively moderate MCP% of the United States, despite its high publication output and central position in collaboration networks, may partly reflect the scale of its domestic research system, which allows substantial collaboration to occur within national boundaries. In contrast, the higher MCP% observed in countries such as Canada, Australia, and Belgium may indicate a greater relative reliance on cross-national research networks; therefore, MCP% should be interpreted as the proportion of internationally coauthored publications rather than as a direct measure of overall collaborative strength. At the same time, the AGS Beers Criteria, STOPP/START criteria, and BZRA deprescribing guidelines have emphasized risk reassessment, gradual tapering, and patient-centered decision-making in older adults [8,9,16,17]. Together, these findings suggest that BZRA deprescribing is increasingly framed not merely as medication discontinuation, but as part of a broader medication-safety and care-management process.

### 4.2. Research Hotspots

By integrating co-citation clustering, keyword co-occurrence analysis, and thematic evolution analysis, this study identified four major research hotspots in BZRA deprescribing among older adults.

#### 4.2.1. BZRA Use and Withdrawal Management

BZRA use and withdrawal management represent one of the earliest evidence-generating topics in this field and remain a core focus of BZRA deprescribing research among older adults. Early studies suggested that long-term benzodiazepine use may be associated with dependence, cognitive impairment, psychomotor dysfunction, and difficulty with withdrawal. In primary care, tapering long-term users requires attention to risk communication, dose adjustment, patient readiness, and continued follow-up [30,31,32]. These concerns provided the basis for subsequent research on how long-term BZRA use can be reduced safely and acceptably in older adults.

Intervention research has examined several approaches to reducing or discontinuing benzodiazepine use. Evidence syntheses suggest that brief interventions, gradual tapering, and psychological support combined with tapering can facilitate discontinuation, although intervention effects vary and evidence for pharmacological substitution remains limited [47]. Pregabalin has also been explored in broader dependence and withdrawal settings [48], but evidence directly applicable to BZRA deprescribing among older adults remains limited. Within the BZRA-specific literature, Vicens et al. demonstrated the value of structured advice and follow-up in primary care; the EMPOWER trial extended this approach through direct patient education; and Reeve et al. synthesized evidence across intervention types while highlighting variation in discontinuation and its durability [29,35,49]. EMPOWER was the most-cited publication in both databases, and Reeve ranked prominently among co-cited authors (Table 3, Table 6 and Table 7), showing that patient education and structured interventions form an important part of the field’s intellectual base.

Consistent with the later citation bursts involving deprescribing guidance and medication-appropriateness criteria (Figure 7A), the guidance literature shows a progressive refinement of withdrawal management. Pottie et al. established a BZRA-specific, evidence-based approach in which gradual tapering among older adults is supported by patient involvement and clinical monitoring [16]. NICE situated benzodiazepine and Z-drug withdrawal within a broader framework for medicines associated with dependence, emphasizing collaborative planning and slow, stepwise dose reduction that can be modified in response to withdrawal symptoms [50]. The 2025 multidisciplinary guideline further developed this approach by placing greater emphasis on ongoing risk–benefit assessment and individualized tapering based on the degree of physical dependence and patient response [17]. Together, these developments reflect the field’s shift from treating discontinuation as a single endpoint toward managing deprescribing as a structured, patient-involved, and adaptive clinical process.

Despite these advances, important uncertainties remain. Existing studies differ substantially in tapering rates, intervention intensity, follow-up duration, and outcome measures. Many studies still use discontinuation rates or dose reduction as primary outcomes, while withdrawal-related outcomes, including rebound insomnia, recurrence of anxiety, withdrawal symptoms, and sustained discontinuation, remain insufficiently assessed. Future research should compare the safety, acceptability, and sustainability of different tapering regimens and identify subgroups of older adults who may benefit from different tapering speeds and support strategies.

#### 4.2.2. Medication Safety and Prescription Optimization in Older Adults

Medication safety and prescription optimization emerged as a major thematic domain in both databases. Polypharmacy ranked fourth among author keywords in both WoSCC and Scopus; potentially inappropriate medications and inappropriate prescribing were prominent in the keyword co-occurrence analyses, while medication review appeared among the recent trend topics. Citation bursts involving updates to the AGS Beers Criteria further indicated sustained attention to medication appropriateness in older adults (Figure 7A, Figure 8 and Figure 9; Table 8). These patterns are consistent with evidence linking higher medication burden to adverse drug reactions, drug–drug interactions, falls, cognitive impairment, hospitalization, and treatment burden [51,52]. Prescription optimization therefore involves reassessing benefits, risks, indications, and treatment burden based on individual clinical needs and patient preferences, rather than simply reducing medication counts [53]. Accordingly, BZRA deprescribing can be situated within the broader framework of geriatric medication review and prescribing appropriateness rather than viewed as an isolated discontinuation decision.

Evidence from studies on polypharmacy and adverse drug reactions supports this perspective. Lavan et al. identified age, comorbidities, medication burden, high-risk drugs, and inadequate monitoring as factors associated with adverse drug reactions in older adults [54]. Halli-Tierney et al. further emphasized identifying medications with unclear indications, duplicate therapies, and potentially inappropriate medications when considering deprescribing [55]. For long-term BZRA users, these findings support evaluating sedative-hypnotic use in the context of the overall medication burden rather than as an isolated prescription issue.

Future research should evaluate how explicit medication appropriateness criteria, including the AGS Beers Criteria and STOPP/START criteria, can be incorporated into structured medication-review pathways for long-term BZRA users [8,9]. Such research may help identify patients requiring closer risk–benefit reassessment, tapering support, or monitoring and inform more consistent approaches across primary care, geriatric care, and pharmacy practice.

#### 4.2.3. Primary Care and Long-Term Care Settings

Primary care and long-term care emerged as prominent care settings in the bibliometric analyses: primary care ranked fifth among author keywords in both WoSCC and Scopus, while nursing home ranked among the ten most frequent keywords in both databases. The WoSCC clustering analysis further identified general practice and drug utilization as themes reflecting clinical context and medication use, respectively, while medication review and quality improvement appeared among the more recent trend topics (Figure 8 and Figure 9; Table 8). Taken together, these patterns suggest that the literature increasingly addresses both care settings and practice-level medication-management processes relevant to BZRA deprescribing. Among these settings, primary care is particularly relevant because long-term BZRA use is often identified through repeat prescriptions, creating opportunities during refill encounters and medication reviews to detect chronic use, reassess the risk–benefit balance, discuss tapering, and arrange follow-up. Broader evidence from a systematic review and meta-analysis indicates that community- and primary-care deprescribing interventions can reduce the use of some potentially inappropriate medications, although effects vary across populations, medication classes, intervention providers, and outcomes [56]. For BZRAs, this evidence supports evaluating whether medication review, patient communication, and follow-up can be integrated into routine refill management, rather than confirming the effectiveness of any specific deprescribing approach.

Pharmacist-supported interventions also formed part of the field’s influential evidence base. The D-PRESCRIBE trial was among the highly cited publications in Scopus and showed a later citation burst in WoSCC, highlighting attention to pharmacist-led patient education and prescriber communication (Figure 7A; Table 7) [36]. Radcliffe et al.’s realist review further identified pharmacist integration, effective team communication, and planned follow-up as mechanisms supporting multidisciplinary medication review and deprescribing in primary care [57]. However, implementation may be constrained by limited consultation time, workload, insufficient knowledge, anticipated patient resistance, and restricted access to nonpharmacological insomnia care such as CBT-I [37,57,58]. These patterns identify the sustainable integration of pharmacist and multidisciplinary support into routine primary care as an area warranting further implementation research.

Long-term care facilities present different implementation challenges. Nursing home residents often have frailty, multimorbidity, complex medication regimens, and exposure to high-risk prescriptions, which make medication review more dependent on routine care processes and staff observation [59]. Staff perceptions of medication necessity, symptom control, and medication-related risks may also shape whether deprescribing is initiated and maintained in these settings [60]. In addition, an updated systematic review and meta-analysis by Quek et al. found that deprescribing interventions generally reduce medication use, but their effects differ by setting, intervention type, and target medication [61]. For BZRAs, this setting-specific evidence suggests that future research should evaluate implementation approaches that integrate medication review with sleep symptom documentation, behavioral symptom assessment, nursing observations, and communication about residents’ care goals.

#### 4.2.4. Patient Acceptance, Nonpharmacological Alternatives, and Safety Outcomes

Patient acceptance is central to implementing BZRA deprescribing. Previous systematic reviews have shown that tapering among older adults is influenced by patients, physicians, nurses, and caregivers. Concerns about insomnia recurrence, withdrawal symptoms, and drug dependence may reduce patients’ willingness to taper, while clinicians’ expectations of resistance may further delay deprescribing initiation [37]. These findings suggest that risk communication alone may be insufficient. Patients’ beliefs about BZRA use, expectations regarding sleep and anxiety control, concerns about symptom recurrence, and preferences for alternative treatments may all influence tapering decisions. Future studies should therefore examine whether shared decision-making and tailored communication can improve acceptance and adherence during deprescribing.

Nonpharmacological support was also represented in the bibliometric findings. The WoSCC clustering analysis identified cognitive behavioral therapy as a distinct cluster, while insomnia and sleep appeared in the keyword and trend analyses (Figure 8C and Figure 9; Table 8). These patterns are clinically relevant because tapering among long-term BZRA users may be accompanied by rebound insomnia, sleep-related anxiety, and reduced confidence in discontinuation. Studies evaluating cognitive behavioral therapy, acceptance and commitment therapy, and mindfulness-based relapse prevention suggest that these approaches may support the management of insomnia and tapering-related concerns [62,63]. The mapped prominence of these topics therefore supports further evaluation of nonpharmacological interventions as components of the deprescribing process rather than solely as treatments introduced after discontinuation.

Safety outcomes are also important when evaluating BZRA deprescribing in older adults. Maust et al. reported that outcomes after benzodiazepine discontinuation among long-term users may be shaped by baseline health status, comorbidities, concomitant medications, and reasons for discontinuation [64]. Accordingly, future studies should not define successful deprescribing solely by discontinuation or dose reduction. In the present bibliometric analysis, terms such as “falls,” “dementia,” “insomnia,” and “sleep” reflected continued attention to geriatric safety and symptom-related outcomes. Future studies should therefore evaluate BZRA deprescribing in relation to both patient-centered outcomes and clinically relevant safety outcomes.

### 4.3. Implications of the Study

The bibliometric patterns identified in this study point to a more implementation-oriented research agenda for BZRA deprescribing in older adults. Future studies should clarify how long-term BZRA users are identified, prioritized, supported, and followed across care settings and determine which components—such as patient education, pharmacist–prescriber communication, behavioral sleep interventions, tapering intensity, and post-taper monitoring—contribute most to safe and acceptable deprescribing. Real-world evidence from primary care, pharmacy practice, and long-term care may further help assess the feasibility and sustainability of these approaches.

More comparable outcome frameworks are also needed. Future studies should assess not only discontinuation or dose reduction but also withdrawal symptoms, rebound insomnia, relapse, sleep quality, anxiety, cognitive function, falls, serious adverse events, quality of life, medication burden, and sustained non-use when relevant. Because implementation may differ across care settings, studies should also report contextual factors such as pharmacist integration, nursing observation, caregiver involvement, and access to behavioral support. Greater standardization of intervention description, patient stratification, and outcome reporting would improve comparability across studies.

Finally, the concentration of research in a limited number of countries and the relatively weak links between major author clusters indicate a need for broader international and cross-team collaboration. Recent evidence also highlights the geographic imbalance of the current evidence base. A 2025 systematic review of BZRA deprescribing interventions found that most included studies were conducted in North America, Europe, or Australia, while evidence from other regions remained limited [65]. At the same time, marked cross-country differences in BZD use and prescribing patterns among older adults, together with emerging longitudinal evidence from Brazil, suggest that prescribing culture, regulatory context, and the availability of medication-review and pharmacist services may influence both BZRA use and the feasibility of deprescribing [66,67]. These geographic and health-system differences may partly explain the relatively sparse links observed between major author clusters, as research teams are often embedded within national or regional care systems with different prescribing practices, regulatory frameworks, professional roles, and implementation priorities. Moreover, because BZRA deprescribing spans geriatrics, primary care, pharmacy, sleep medicine, and long-term care, sustained cross-team collaboration requires coordination across disciplinary and healthcare-system boundaries, which may create additional organizational barriers to developing stable multinational research networks. Future studies should include more diverse healthcare systems, particularly underrepresented low- and middle-income regions, and evaluate whether deprescribing models are transferable across different clinical and policy contexts. Digital approaches, including electronic clinical decision support, digital CBT-I, and AI-assisted medication review, also warrant evaluation as potential tools to improve the scalability of BZRA deprescribing [7,68].

### 4.4. Limitations

This study has several limitations. First, only WoSCC and Scopus were included. However, these two databases are widely used in bibliometric research and cover a large body of the medical, pharmaceutical, and health science literature; relevant studies indexed in PubMed, Embase, PsycINFO, CINAHL, and regional databases may have been omitted. Therefore, the findings primarily reflect the international research landscape captured by WoSCC and Scopus and may not fully represent the entire body of literature on BZRA deprescribing among older adults.

Second, this study included only English-language articles and reviews, which may have introduced language and publication-type bias. Some studies from non-English-speaking countries or regions on BZRA use, deprescribing practices, primary care prescribing management, or long-term care medication review may have been published in local-language journals or gray literature and were not included. This may have led to an underestimation of research activity and practice-based contributions from China, other Asian countries, and low- and middle-income countries.

Third, bibliometric analysis can reveal publication output, collaboration patterns, intellectual structures, and thematic evolution, but it cannot directly assess the clinical effectiveness, safety, or quality of evidence for specific deprescribing interventions. For example, keyword and co-citation analyses can indicate that CBT-I, pharmacist involvement, patient education, and long-term care settings are important topics. However, they cannot determine the actual effectiveness of these interventions across different populations, healthcare systems, or care settings. Therefore, future research should combine systematic reviews, meta-analyses, randomized controlled trials, implementation studies, and real-world evidence to further evaluate the clinical value, feasibility, and sustainability of different BZRA deprescribing pathways.

Fourth, although many core bibliometric analyses were conducted in both WoSCC and Scopus, some analyses were database-specific. CiteSpace-based analyses, the dual-map overlay, and the keyword clustering analysis were based on WoSCC because of differences in database structure and software compatibility. Therefore, these findings should be interpreted as complementary to, rather than directly equivalent to, the analyses performed across both databases.

## 5. Conclusions

Using WoSCC and Scopus, this study systematically mapped the global research landscape, intellectual structure, and thematic evolution of BZRA deprescribing among older adults from 2008 to May 2026, with 2026 treated as a partial year. Publication output showed an overall upward trend, with output during 2021–2025 generally higher than in preceding years. The research focus gradually expanded from withdrawal and discontinuation toward medication safety, prescription optimization, patient engagement, nonpharmacological management, and implementation in primary care and long-term care settings.

Keyword, co-citation, and trend analyses revealed that BZRA deprescribing is no longer limited to reducing or discontinuing a single class of medications but has increasingly been considered within a broader framework of medication safety and prescription optimization. These findings suggest that BZRA deprescribing is progressively shifting from an isolated medication reduction strategy toward a broader framework of geriatric medication management. Future research should prioritize the comparative evaluation of tapering strategies, nonpharmacological support, multidisciplinary care approaches, and standardized long-term safety outcomes. The increasing attention to medication review and deprescribing implementation across primary care and long-term care settings may provide insights for developing and evaluating scalable approaches to support appropriate BZRA use. Emerging digital approaches, including electronic clinical decision support, digital CBT-I, and AI-assisted medication review, also warrant further evaluation as potential tools to facilitate implementation.

Overall, BZRA deprescribing research has evolved beyond the question of whether medications should be discontinued toward a more careful consideration of how medication safety, patient needs, and care systems can be integrated to support appropriate medication use among older adults.

## Figures and Tables

**Table 1:** Publication output and international collaboration according to corresponding-author country in WoSCC and Scopus. (**A**) Publication output and international collaboration by corresponding-author country. (**B**) Country contributions based on all author affiliations using full and fractional counting.

(A)					
Country/Region	Publications (*n*)	SCP (*n*)		MCP (*n*)	MCP Ratio (%)
WoSCC					
United States	73	64		9	12.3
Canada	41	34		7	17.1
Australia	30	22		8	26.7
Belgium	21	14		7	33.3
France	20	13		7	35.0
Spain	14	11		3	21.4
United Kingdom	11	6		5	45.5
Germany	9	9		0	0.0
Denmark	8	8		0	0.0
Japan	8	5		3	37.5
**Scopus**					
United States	103	89		14	13.6
Canada	50	38		12	24.0
Australia	33	26		7	21.2
France	23	18		5	21.7
Belgium	21	11		10	47.6
Japan	15	12		3	20.0
Spain	15	11		4	26.7
Ireland	12	10		2	16.7
Germany	11	10		1	9.1
United Kingdom	9	5		4	44.4
**(B)**					
All-author fractional publication count ranks countries/regions.					
**Country/Region**	**All-Author Full-Count** **Publications**		**All-Author Fractional-Count** **Publications**		**Fractional Rank**
**WoSCC**					
United States	89		75.22		1
Canada	56		42.94		2
Australia	33		27.33		3
Belgium	26		17.72		4
France	23		17.27		5
United Kingdom	21		14.13		6
Spain	19		12.83		7
Germany	11		9.60		8
Switzerland	17		8.55		9
Norway	13		7.44		10
**Scopus**					
United States	130		111.00		1
Canada	78		56.86		2
Australia	47		37.29		3
France	29		22.18		4
United Kingdom	25		16.84		5
Belgium	26		16.16		6
Japan	18		16.00		7
Spain	25		15.89		8
Ireland	16		11.47		9
Switzerland	21		11.35		10

SCP, single-country publication; MCP, multiple-country publication. (**A**) is based on the country/region of the corresponding author. (**B**) is based on all-author affiliations. Under full counting, each country/region represented in a publication received one credit; under fractional counting, a publication involving k countries/regions contributed 1/k to each country/region.

**Table 2:** Most productive affiliations in WoSCC and Scopus.

WoSCC					Scopus			
Rank	Institution	Publications	Degree	Weighted Degree	Institution	Publications	Degree	Weighted Degree
1	University of Montreal	19	8	12	University of Montreal	18	13	15
2	University of Toronto	15	11	11	University of Toronto	15	12	11
3	Harvard Medical School	14	6	13	UCLouvain	15	4	6
4	UCLouvain	14	9	7	University of North Carolina at Chapel Hill	15	7	6
5	University of Sydney	14	3	6	McGill University	13	11	12
6	University of Ottawa	12	16	12	University of Sydney	13	5	5
7	University of Bern	11	14	9	University of Ottawa	12	14	12
8	University of Michigan	10	3	7	Dalhousie University	12	12	7
9	University of Oslo	10	7	5	University of Bern	11	7	8
10	Dalhousie University	10	9	4	University of Oslo	11	3	4

Degree, number of direct collaboration links; weighted degree, total strength of collaboration links.

**Table 3:** Top authors and co-cited authors in WoSCC and Scopus.

Rank	Authors	Publications	Citations	Co-Cited Authors	Co-Citations
WoSCC					
1	Spinewine A	16	151	Reeve E	167
2	Tannenbaum C	12	873	Tannenbaum C	113
3	Henrard S	10	83	Martin P	88
4	Martin P	7	753	Fick DM	73
5	Kivelä SL	7	168	Morin CM	69
6	Vahlberg T	7	168	Glass J	67
7	Evrard P	7	96	Vicens C	66
8	Puustinen J	6	123	Voshaar RCO	65
9	Pétein C	6	74	Lader M	63
10	Maust DT	6	62	O’Mahony D	59
**Scopus**					
1	Spinewine A	16	172	Hilmer SN	104
2	Tannenbaum C	11	1159	Tannenbaum C	101
3	Niznik JD	11	134	Reeve E	86
4	Henrard S	10	94	Ahmed S	79
5	Turner JP	8	119	O’Mahony D	63
6	Tamblyn R	7	1034	Lader M	60
7	Hilmer SN	7	339	Wiese MD	53
8	Kivelä SL	7	190	Gnjidic D	51
9	Vahlberg T	7	190	Le Couteur DG	42
10	Evrard P	7	108	Zitman FG	41

**Table 4:** Top 10 sources by number of publications in WoSCC and Scopus.

WoSCC					Scopus			
Rank	Source	Documents	TC	CPD	Source	Documents	TC	CPD
1	*Journal of the American Geriatrics Society*	23	323	14.04	*Journal of the American Geriatrics Society*	32	599	18.72
2	*Drugs & Aging*	17	686	40.35	*Drugs & Aging*	17	727	42.76
3	*BMC Geriatrics*	12	180	15.00	*Journal of the American Medical Directors Association*	13	266	20.46
4	*BMJ Open*	11	228	20.73	*European Journal of Clinical Pharmacology*	12	310	25.83
5	*European Journal of Clinical Pharmacology*	10	315	31.50	*BMJ Open*	11	345	31.36
6	*Journal of the American Medical Directors Association*	8	273	34.13	*BMC Geriatrics*	10	170	17.00
7	*PLOS ONE*	7	212	30.29	*JAMA Network Open*	9	124	13.78
8	*British Journal of Clinical Pharmacology*	6	335	55.83	*Age and Ageing*	8	577	72.13
9	*JAMA Network Open*	6	110	18.33	*British Journal of Clinical Pharmacology*	7	370	52.86
10	*Journal of General Internal Medicine*	6	66	11.00	*Research in Social and Administrative Pharmacy*	7	87	12.43

TC, total citations; CPD, citations per document.

**Table 5:** Top 10 sources by total citations in WoSCC and Scopus.

WoSCC					Scopus			
Rank	Source	TC	Documents	CPD	Source	TC	Documents	CPD
1	*Drugs & Aging*	686	17	40.35	*Drugs & Aging*	727	17	42.76
2	*JAMA Internal Medicine*	525	3	175.00	*Journal of the American Geriatrics Society*	599	32	18.72
3	*Addiction*	467	2	233.50	*Age and Ageing*	577	8	72.13
4	*Age and Ageing*	438	5	87.60	*JAMA Internal Medicine*	557	3	185.67
5	*British Journal of Clinical Pharmacology*	335	6	55.83	*Mayo Clinic Proceedings*	386	2	193.00
6	*Journal of the American Geriatrics Society*	323	23	14.04	*British Journal of Clinical Pharmacology*	370	7	52.86
7	*European Journal of Clinical Pharmacology*	315	10	31.50	*BMJ Open*	345	11	31.36
8	*Canadian Family Physician*	298	2	149.00	*JAMA*	325	1	325.00
9	*Journal of the American Medical Directors Association*	273	8	34.13	*European Journal of Clinical Pharmacology*	310	12	25.83
10	*CNS Drugs*	246	3	82.00	*CNS Drugs*	303	2	151.50

**Table 6:** Top 10 most-cited documents in WoSCC.

WoSCC			
Paper	Title	Total Citations	TC Per Year
TANNENBAUM C, 2014, JAMA INTERN MED	Reduction of Inappropriate Benzodiazepine Prescriptions Among Older Adults Through Direct Patient Education: The EMPOWER Cluster Randomized Trial.	495	38.08
LADER M, 2011, ADDICTION	Benzodiazepines revisited—will we ever learn?	412	25.75
CLEGG A, 2011, AGE AGEING	Which medications to avoid in people at risk of delirium: a systematic review	382	23.88
IYER S, 2008, DRUG AGING	Medication withdrawal trials in people aged 65 years and older: a systematic review	269	14.16
LADER M, 2014, BRIT J CLIN PHARMACO	Benzodiazepine harm: how can it be reduced?	233	17.92
POTTIE K, 2018, CAN FAM PHYSICIAN	Deprescribing benzodiazepine receptor agonists: Evidence-based clinical practice guideline	229	25.44
LADER M, 2009, CNS DRUGS	Withdrawing benzodiazepines in primary care	185	10.28
REEVE E, 2017, EUR J CLIN PHARMACOL	A systematic review of interventions to deprescribe benzodiazepines and other hypnotics among older people	143	14.30
HOEL RW, 2021, MAYO CLIN PROC	Polypharmacy management in older patients	142	23.67
FARRELL B, 2015, PLOS ONE	What are priorities for deprescribing for elderly patients? Capturing the voice of practitioners: a modified delphi process	134	11.17

**Table 7:** Top 10 most-cited documents in Scopus.

Scopus			
Paper	Title	Total Citations	TC Per Year
TANNENBAUM C, 2014, JAMA INTERN MED	Reduction of inappropriate benzodiazepine prescriptions among older adults through direct patient education: the EMPOWER cluster randomized trial	531	40.85
CLEGG A, 2011, AGE AGEING	Which medications to avoid in people at risk of delirium: a systematic review	480	30.00
MARTIN P, 2018, JAMA	Effect of a pharmacist-led educational intervention on inappropriate medication prescriptions in older adults: the D-PRESCRIBE randomized clinical trial	325	36.11
IYER S, 2008, DRUGS AGING	Medication withdrawal trials in people aged 65 years and older: a systematic review	319	16.79
LADER M, 2014, BR J CLIN PHARMACOL	Benzodiazepine harm: how can it be reduced?	261	20.08
POTTIE K, 2018, CAN FAM PHYS	Deprescribing benzodiazepine receptor agonists: Evidence-based clinical practice guideline	260	28.89
MARKOTA M, 2016, MAYO CLIN PROC	Benzodiazepine Use in Older Adults: Dangers, Management, and Alternative Therapies	231	21.00
LADER M, 2009, CNS DRUGS	Withdrawing Benzodiazepines in Primary Care	226	12.56
BRETT J, 2015, AUST PRESCR	Management of benzodiazepine misuse and dependence	206	17.17
REEVE E, 2017, EUR J CLIN PHARMACOL	A systematic review of interventions to deprescribe benzodiazepines and other hypnotics among older people	157	15.70

**Table 8:** Top 20 most frequent keywords in WoSCC and Scopus.

WoSCC			Scopus	
Rank	Author Keyword	Occurrences	Author Keyword	Occurrences
1	benzodiazepines	107	benzodiazepines	132
2	older adults	91	deprescribing	127
3	deprescribing	77	older adults	119
4	polypharmacy	32	polypharmacy	67
5	primary care	30	primary care	35
6	z-drugs	19	potentially inappropriate medications	25
7	insomnia	18	dementia	24
8	potentially inappropriate medications	18	inappropriate prescribing	20
9	inappropriate prescribing	17	nursing home	20
10	nursing home	16	z-drugs	18
11	hypnotics	15	falls	17
12	dementia	14	geriatrics	17
13	zolpidem	14	insomnia	17
14	discontinuation	13	zolpidem	15
15	falls	13	hypnotics	14
16	drug utilization	6	opioids	13
17	geriatrics	6	discontinuation	10
18	patient education	6	geriatric medicine	9
19	pharmacoepidemiology	6	pharmacoepidemiology	9
20	sleep	6	antipsychotic	7

## Data Availability

No new data were created or analyzed in this study. Data sharing is not applicable to this article.

## Supplementary Materials

The following supporting information can be downloaded at: https://www.mdpi.com/article/10.3390/healthcare14172856/s1, Table S1: Search strategies in the Web of Science Core Collection and Scopus; Table S2: Data standardization and thesaurus rules used in bibliometric analyses; Table S3: Completed BIBLIO checklist for reporting this bibliometric review; Table S4: Publications ranked 1–20 by total citations in WoSCC; Table S5: Publications ranked 1–20 by total citations in Scopus.

## Abbreviations

The following abbreviations are used in this manuscript:

AGS	American Geriatrics Society
BZRA	Benzodiazepine receptor agonist
BZRAs	Benzodiazepine receptor agonists
CBT-I	Cognitive behavioral therapy for insomnia
IF	Impact Factor
JCR	Journal Citation Reports
MCP	Multiple-country publications
MCP%	Proportion of multiple-country publications
R^2^	Coefficient of determination
TC	Total citations
WoSCC	Web of Science Core Collection
