# Supplementary material for: Global Trends in Deprescribing Benzodiazepine Receptor Agonists in Older Adults: A Dual-Database Bibliometric and Visual Analysis
Source: Healthcare (Basel). 2026 Sep 4;14(17):2856. doi: 10.3390/healthcare14172856 (PMC13565376; doi:10.3390/healthcare14172856)
Supplement: Supplementary file 1 [file healthcare-14-02856-s001.zip › healthcare-4438787-supplementary.pdf]

Supplementary Materials

Table S1. Search strategies in the Web of Science Core Collection and Scopus.

Database	Step	Search concept	Search expression
WoSCC	#1	Older adults	TS=(elderly OR geriatric* OR "older adult*" OR "older patient*" OR "older person*" OR "older people" OR "older individual*" OR "aged 65 year*" OR "65 years or older" OR "65 years and older" OR "over 65")
WoSCC	#2	Benzodiazepine receptor agonists and related hypnotics	TS=("benzodiazepine receptor agonist*" OR BZRA* OR benzodiazepin* OR "z-drug*" OR "Z-drug*" OR "z drug*" OR "Z drug*" OR "z-hypnotic*" OR "z hypnotic*" OR "nonbenzodiazepine hypnotic*" OR "non-benzodiazepine hypnotic*" OR "benzodiazepine-like hypnotic*" OR "benzodiazepine like hypnotic*" OR zolpidem OR zopiclone OR eszopiclone OR zaleplon OR temazepam OR triazolam OR estazolam OR nitrazepam OR flurazepam OR quazepam OR brotizolam OR lormetazepam)
WoSCC	#3	Deprescribing, discontinuation, tapering, withdrawal, or dose reduction	TS=(deprescrib* OR "de-prescrib*" OR unprescrib* OR ((discontinu* OR reduc* OR stop* OR taper* OR withdraw* OR cessation* OR "dose reduc*" OR "dose reduction") NEAR/5 ("benzodiazepine receptor agonist*" OR BZRA* OR benzodiazepin* OR "z-drug*" OR "Z-drug*" OR "z drug*" OR "Z drug*" OR "z-hypnotic*" OR "z hypnotic*" OR "nonbenzodiazepine hypnotic*" OR "non-benzodiazepine hypnotic*" OR "benzodiazepine-like hypnotic*" OR "benzodiazepine like hypnotic*" OR zolpidem OR zopiclone OR eszopiclone OR zaleplon OR temazepam OR triazolam OR estazolam OR nitrazepam OR flurazepam OR quazepam OR brotizolam OR lormetazepam)))
WoSCC	#4	Final combined query	#1 AND #2 AND #3
Scopus	#1	Older adults	TITLE-ABS-KEY(elderly OR geriatric* OR "older adult*" OR "older patient*" OR "older person*" OR "older people" OR "older individual*" OR "aged 65 year*" OR "65 years or older" OR "65 years and older" OR "over 65")
Scopus	#2	Benzodiazepine receptor agonists and related hypnotics	TITLE-ABS-KEY("benzodiazepine receptor agonist*" OR BZRA* OR benzodiazepin* OR "z-drug*" OR "Z-drug*" OR "z drug*" OR "Z drug*" OR "z-hypnotic*" OR "z hypnotic*" OR "nonbenzodiazepine hypnotic*" OR "non-benzodiazepine hypnotic*" OR "benzodiazepine-like hypnotic*" OR "benzodiazepine like hypnotic*" OR zolpidem OR zopiclone OR eszopiclone OR zaleplon OR temazepam OR triazolam OR estazolam OR nitrazepam OR flurazepam OR quazepam OR brotizolam OR lormetazepam)
Scopus	#3	Deprescribing, discontinuation, tapering, withdrawal, or dose reduction	TITLE-ABS-KEY(deprescrib* OR "de-prescrib*" OR unprescrib* OR ((discontinu* OR reduc* OR stop* OR taper* OR withdraw* OR cessation* OR "dose reduc*" OR "dose reduction") W/5 ("benzodiazepine receptor agonist*" OR BZRA* OR benzodiazepin* OR "z-drug*" OR "Z-drug*" OR "z drug*" OR "Z drug*" OR "z-hypnotic*" OR "z hypnotic*" OR "nonbenzodiazepine hypnotic*" OR "non-benzodiazepine hypnotic*" OR "benzodiazepine-like hypnotic*" OR "benzodiazepine like hypnotic*" OR zolpidem OR zopiclone OR eszopiclone OR zaleplon OR temazepam OR triazolam OR estazolam OR nitrazepam OR flurazepam OR quazepam OR brotizolam OR lormetazepam)))
Scopus	#4	Final combined query	#1 AND #2 AND #3

WoSCC, Web of Science Core Collection; TS, Topic Search; TITLE-ABS-KEY, title, abstract, and keywords search in Scopus.

Table S2. Data standardization and thesaurus rules used in bibliometric analyses.

The same standardization logic was applied to the WoSCC and Scopus datasets before visualization and trend analyses. Original labels are shown as they appeared in the thesaurus files. "[excluded]" indicates generic or non-informative labels removed from the relevant analyses.

Table S2A. Institution-name merge rules.

Original label(s)	Standardized label	Action	Rationale
beth israel deaconess med ctr; beth israel deaconess medical center	Beth Israel Deaconess Medical Center	Merge	Institution-name abbreviation, spelling variant, or sub-unit variant
boston univ; boston university	Boston University	Merge	Institution-name abbreviation, spelling variant, or sub-unit variant
brigham & womens hosp; brigham and women's hospital; brigham and womens hospital	Brigham and Women's Hospital	Merge	Institution-name abbreviation, spelling variant, or sub-unit variant
cedars sinai med ctr; cedars sinai medical center; cedars-sinai medical center	Cedars-Sinai Medical Center	Merge	Institution-name abbreviation, spelling variant, or sub-unit variant
center for addiction and mental health; centre addict & mental hlth; centre for addiction and mental health; ctr addict & mental hlth	Centre for Addiction and Mental Health	Merge	Institution-name abbreviation, spelling variant, or sub-unit variant
charles univ; charles university	Charles University	Merge	Institution-name abbreviation, spelling variant, or sub-unit variant
chu ucl namur	CHU UCL Namur	Merge	Institution-name abbreviation, spelling variant, or sub-unit variant
dalhousie univ; dalhousie university	Dalhousie University	Merge	Institution-name abbreviation, spelling variant, or sub-unit variant
duke univ; duke university; duke university school medi; duke university school of medi; duke university school of medicine	Duke University	Merge	Institution-name abbreviation, spelling variant, or sub-unit variant
ghent university; univ ghent	Ghent University	Merge	Institution-name abbreviation, spelling variant, or sub-unit variant
ghent univ hosp; ghent university hospital	Ghent University Hospital	Merge	Institution-name abbreviation, spelling variant, or sub-unit variant
harvard med sch; harvard medical school	Harvard Medical School	Merge	Institution-name abbreviation, spelling variant, or sub-unit variant
institute of health and societ; institute of health and society	Institute of Health and Society	Merge	Institution-name abbreviation, spelling variant, or sub-unit variant
johns hopkins univ; johns hopkins university; johns hopkins university schoo; johns hopkins university school; johns hopkins university school of medicine	Johns Hopkins University	Merge	Institution-name abbreviation, spelling variant, or sub-unit variant
kaiser permanente washington h; kaiser permanente washington health; kaiser permanente washington health research institute	Kaiser Permanente Washington Health Research Institute	Merge	Institution-name abbreviation, spelling variant, or sub-unit variant
laval university; univ laval; universite laval; université laval	Laval University	Merge	Institution-name abbreviation, spelling variant, or sub-unit variant
louvain drug research institut; louvain drug research institute	Louvain Drug Research Institute	Merge	Institution-name abbreviation, spelling variant, or sub-unit variant
marcus institute for aging res; marcus institute for aging research	Marcus Institute for Aging Research	Merge	Institution-name abbreviation, spelling variant, or sub-unit variant
massachusetts general hosp; massachusetts general hospital	Massachusetts General Hospital	Merge	Institution-name abbreviation, spelling variant, or sub-unit variant
mcgill univ; mcgill university	McGill University	Merge	Institution-name abbreviation, spelling variant, or sub-unit variant
mcgill university health center; mcgill university health centr; mcgill university health centre	McGill University Health Centre	Merge	Institution-name abbreviation, spelling variant, or sub-unit variant
mcmaster univ; mcmaster university	McMaster University	Merge	Institution-name abbreviation, spelling variant, or sub-unit variant
monash univ; monash university	Monash University	Merge	Institution-name abbreviation, spelling variant, or sub-unit variant
oslo univ hosp; oslo university hospital	Oslo University Hospital	Merge	Institution-name abbreviation, spelling variant, or sub-unit variant
ottawa hosp; ottawa hospital	Ottawa Hospital	Merge	Institution-name abbreviation, spelling variant, or sub-unit variant
queen's university; queens university	Queen's University	Merge	Institution-name abbreviation, spelling variant, or sub-unit variant
royal north shore hosp; royal north shore hospital	Royal North Shore Hospital	Merge	Institution-name abbreviation, spelling variant, or sub-unit variant
stanford univ; stanford university; stanford university school of medicine	Stanford University	Merge	Institution-name abbreviation, spelling variant, or sub-unit variant
catholic univ louvain; catholic university of louvain; louvain univ; u c louvain; uclouvain; universite catholique de louva; universite catholique de louvain; university catholic louvain; université catholique de louva; université catholique de louvain	UCLouvain	Merge	Institution-name abbreviation, spelling variant, or sub-unit variant
university college cork	University College Cork	Merge	Institution-name abbreviation, spelling variant, or sub-unit variant
univ adelaide; university of adelaide	University of Adelaide	Merge	Institution-name abbreviation, spelling variant, or sub-unit variant
univ alberta; university of alberta	University of Alberta	Merge	Institution-name abbreviation, spelling variant, or sub-unit variant
univ antwerp; university of antwerp	University of Antwerp	Merge	Institution-name abbreviation, spelling variant, or sub-unit variant
univ bern; university of bern	University of Bern	Merge	Institution-name abbreviation, spelling variant, or sub-unit variant
univ british columbia; university of british columbia	University of British Columbia	Merge	Institution-name abbreviation, spelling variant, or sub-unit variant
univ calgary; university of calgary	University of Calgary	Merge	Institution-name abbreviation, spelling variant, or sub-unit variant
univ california; university of california	University of California	Merge	Institution-name abbreviation, spelling variant, or sub-unit variant
ucla; univ calif los angeles; university of california los angeles	University of California Los Angeles	Merge	Institution-name abbreviation, spelling variant, or sub-unit variant
ucsf; univ calif san francisco; university of california san francisco	University of California San Francisco	Merge	Institution-name abbreviation, spelling variant, or sub-unit variant
univ colorado; university of colorado	University of Colorado	Merge	Institution-name abbreviation, spelling variant, or sub-unit variant
univ copenhagen; university of copenhagen	University of Copenhagen	Merge	Institution-name abbreviation, spelling variant, or sub-unit variant
univ massachusetts; university of massachusetts	University of Massachusetts	Merge	Institution-name abbreviation, spelling variant, or sub-unit variant
univ michigan; university of michigan	University of Michigan	Merge	Institution-name abbreviation, spelling variant, or sub-unit variant
univ montreal; universite de montreal; universite de montréal; university of montreal; université de montreal; université de montréal	University of Montreal	Merge	Institution-name abbreviation, spelling variant, or sub-unit variant

Original label(s)	Standardized label	Action	Rationale
univ north carolina; univ north carolina a; university of north carolina; university of north carolina a	University of North Carolina	Merge	Institution-name abbreviation, spelling variant, or sub-unit variant
univ oslo; university of oslo	University of Oslo	Merge	Institution-name abbreviation, spelling variant, or sub-unit variant
univ ottawa; university of ottawa	University of Ottawa	Merge	Institution-name abbreviation, spelling variant, or sub-unit variant
univ pittsburgh; university of pittsburgh	University of Pittsburgh	Merge	Institution-name abbreviation, spelling variant, or sub-unit variant
univ sherbrooke; universite de sherbrooke; university of sherbrooke; université de sherbrooke	University of Sherbrooke	Merge	Institution-name abbreviation, spelling variant, or sub-unit variant
univ south australia; university of south australia	University of South Australia	Merge	Institution-name abbreviation, spelling variant, or sub-unit variant
the university of sydney; univ sydney; university of sydney; university sydney	University of Sydney	Merge	Institution-name abbreviation, spelling variant, or sub-unit variant
univ toronto; university of toronto	University of Toronto	Merge	Institution-name abbreviation, spelling variant, or sub-unit variant
univ tsukuba; university of tsukuba	University of Tsukuba	Merge	Institution-name abbreviation, spelling variant, or sub-unit variant
univ washington; university of washington	University of Washington	Merge	Institution-name abbreviation, spelling variant, or sub-unit variant
univ waterloo; university of waterloo	University of Waterloo	Merge	Institution-name abbreviation, spelling variant, or sub-unit variant
univ zagreb; university of zagreb	University of Zagreb	Merge	Institution-name abbreviation, spelling variant, or sub-unit variant
va ann arbor healthcare syst; va ann arbor healthcare system	VA Ann Arbor Healthcare System	Merge	Institution-name abbreviation, spelling variant, or sub-unit variant

Table S2B. Country/region-name standardization rules.

Original label(s)	Standardized label	Action	Rationale
bolivia, plurinational state of	bolivia	Merge	Country/region naming variant or abbreviation
brunei darussalam	brunei	Merge	Country/region naming variant or abbreviation
china mainland; mainland china; p r china; p.r.c; p.r.c.; people s r china; people s republic of china; people's r china; people's republic of china; peoples r china; peoples republic of china; pr china; prc	china	Merge	Country/region naming variant or abbreviation
cote d'ivoire; côte d'ivoire; côte d’ivoire	cote d'ivoire	Merge	Country/region naming variant or abbreviation
czech rep; czech rep.; czechia	czech republic	Merge	Country/region naming variant or abbreviation
iran, islamic republic of; islamic republic of iran	iran	Merge	Country/region naming variant or abbreviation
korea republic of; korea, republic of; republic of korea; s korea; south korea	korea	Merge	Country/region naming variant or abbreviation
lao pdr; lao people's democratic republic	laos	Merge	Country/region naming variant or abbreviation
moldova, republic of	moldova	Merge	Country/region naming variant or abbreviation
holland; the netherlands	netherlands	Merge	Country/region naming variant or abbreviation
russian federation	russia	Merge	Country/region naming variant or abbreviation
slovak republic	slovakia	Merge	Country/region naming variant or abbreviation
syrian arab republic	syria	Merge	Country/region naming variant or abbreviation
tanzania, united republic of; united republic of tanzania	tanzania	Merge	Country/region naming variant or abbreviation
turkiye; türkiye	turkey	Merge	Country/region naming variant or abbreviation
britain; england; great britain; northern ireland; scotland; u.k; u.k.; uk; wales	united kingdom	Merge	Country/region naming variant or abbreviation
america; u.s; u.s.; u.s.a; u.s.a.; united states of america; us; usa	united states	Merge	Country/region naming variant or abbreviation
venezuela, bolivarian republic of	venezuela	Merge	Country/region naming variant or abbreviation
viet nam	vietnam	Merge	Country/region naming variant or abbreviation

Table S2C. Keyword merge rules.

Original label(s)	Standardized label	Action	Rationale
antipsychotics	antipsychotic	Merge	Keyword abbreviation, spelling variant, plural form, or synonym
beer criteria; beer's criteria; beer's criterion; beers criteria; beers criterion	Beers criteria	Merge	Keyword abbreviation, spelling variant, plural form, or synonym
benzodiazepine receptor agonist; bzra; bzras	benzodiazepine receptor agonists	Merge	Keyword abbreviation, spelling variant, plural form, or synonym
benzodiazepine; benzodiazepine use; benzodiazepine users; bzd; bzds	benzodiazepines	Merge	Keyword abbreviation, spelling variant, plural form, or synonym
de prescribing; de-prescribing; deprescribe; deprescribed; deprescription; deprescriptions	deprescribing	Merge	Keyword abbreviation, spelling variant, plural form, or synonym
drug utilisation	drug utilization	Merge	Keyword abbreviation, spelling variant, plural form, or synonym
accidental fall; accidental falls; fall	falls	Merge	Keyword abbreviation, spelling variant, plural form, or synonym
hypnotic; sedative hypnotic; sedative hypnotics; sedative-hypnotic; sedative-hypnotics	hypnotics	Merge	Keyword abbreviation, spelling variant, plural form, or synonym
inappropriate prescription; inappropriate prescriptions	inappropriate prescribing	Merge	Keyword abbreviation, spelling variant, plural form, or synonym
long term care; longterm care	long-term care	Merge	Keyword abbreviation, spelling variant, plural form, or synonym
medication reviews	medication review	Merge	Keyword abbreviation, spelling variant, plural form, or synonym
nursing homes	nursing home	Merge	Keyword abbreviation, spelling variant, plural form, or synonym
aged; elderly; geriatric patient; geriatric patients; older adult; older patient; older patients; older people; older person; older persons	older adults	Merge	Keyword abbreviation, spelling variant, plural form, or synonym
pim; pims; potentially inappropriate medication; potentially inappropriate medicine; potentially inappropriate medicines	potentially inappropriate medications	Merge	Keyword abbreviation, spelling variant, plural form, or synonym
general practice; general practitioner; general practitioners; primary health care; primary healthcare	primary care	Merge	Keyword abbreviation, spelling variant, plural form, or synonym
non-benzodiazepine hypnotic; non-benzodiazepine hypnotics; nonbenzodiazepine hypnotic; nonbenzodiazepine hypnotics; z drug; z drugs; z-drug; z-drugs	z-drugs	Merge	Keyword abbreviation, spelling variant, plural form, or synonym

Table S2D. Exclusion rules for generic or non-informative labels.

Category	Original label(s)	Action	Rationale
Institution: department-level terms	department of anesthesiology; department of biostatistics; department of clinical pharmacology; department of epidemiology; department of family medicine; department of geriatric medicine; department of internal medicine; department of medicine; department of neurology; department of pharmacology; department of pharmacy; department of primary care; department of psychiatry; department of psychiatry and behavioral sciences; department of psychiatry and psychology; department of psychology; department of public health; department of social pharmacy	Exclude	Generic affiliation term without identifiable institutional information
Institution: division-level terms	division of general medicine; division of geriatric medicine; division of geriatrics; division of pharmacology and p; division of social pharmacy	Exclude	Generic affiliation term without identifiable institutional information
Institution: faculty/school/college-level terms	college of health sciences; college of pharmacy; college of public affairs; faculty of medicine; faculty of nursing; faculty of pharmacy; graduate school of medicine; school of medicine; school of nursing; school of pharmacy; school of public health	Exclude	Generic affiliation term without identifiable institutional information
Institution: other generic affiliation terms	leslie dan faculty of pharmacy; medical sciences campus; medical teaching and research; temerty faculty of medicine	Exclude	Generic affiliation term without identifiable institutional information
Keyword	prescribing; prescriptions; systematic review	Exclude	Overly generic, non-thematic, or document-type term

Table S3. Completed BIBLIO checklist for reporting this bibliometric review.

Section/Topic	Item No.	Checklist item	Reported location in the manuscript
**Title**			
Identification	1	Identify the report as a bibliometric review or bibliometric analysis in the title.	Title page, page 1 (title identifies the study as a dual-database bibliometric and visual analysis).
Issues/topics	2	Indicate the key issue/topic under investigation and the coverage of the time period.	Topic: Title page and Highlights, pages 1–2. Time coverage: Abstract, page 2, and Section 2.1, page 5 (1 January 2008 to 21 May 2026; 2026 treated as a partial year).
**Abstract**			
Structured summary	3	Provide a structured summary including background/objectives, methods, results, key findings, and conclusions.	Abstract, page 2 (structured summary: Objectives, Methods, Results, and Conclusions).
**Introduction/Background**			
Justification/rationale/explanation	4	Present the existing knowledge, rationale, and background information relevant to the topic.	Introduction, pages 3–4 (BZRA-related risks, clinical guidance, deprescribing rationale, bibliometric research gap, and dual-database rationale).

Section/Topic	Item No.	Checklist item	Reported location in the manuscript
Objectives	5	State the objective(s) or research question(s) of the bibliometric review.	Introduction, final paragraph, pages 4–5 (objectives: mapping publication trends, contributors, collaboration patterns, knowledge structures, and research hotspots).
**Methods**			
Search engines/data sources	6	Describe all information sources and databases used for literature retrieval.	Section 2.1, page 5 (WoSCC and Scopus as two independent data sources; final search date reported).
Search strategy	7	Report the search terms and search criteria, including search date, language, document type, and database-specific search strategy.	Sections 2.2–2.3, pages 5–6 (eligibility, search fields, Boolean/proximity logic, screening, and data export); Supplementary Table S1 (complete WoSCC and Scopus search strings).
Time period	8	Report the time period covered by the review and provide the justification or context for this period.	Introduction, page 4 (rationale for retaining 2008 as the pragmatic starting year); Section 2.1, page 5 (review period and final search date); Section 2.4, pages 7–8 (2026 treated as incomplete and excluded from complete-year trend models).
Eligibility criteria	9	Describe inclusion and exclusion criteria, including language, study design, document type, publication type, and time period.	Section 2.2, page 5 (English-language articles/reviews, age criterion, BZRA/deprescribing eligibility criteria, mixed-age rules, and excluded document types).
Data refinement/data selection procedure	10	Describe the process used to remove irrelevant records and to screen records based on title, abstract, and content.	Section 2.3, pages 5–6 (independent screening, topical relevance assessment, full-text review when required, database-specific data export, and cross-database overlap assessment); Figure 1, page 8 (workflow and overlap).
Quality assessment (optional)	11	Report quality assessment if performed.	Not applicable. This bibliometric review analyzed bibliographic records rather than primary clinical studies; no formal study-level quality or risk-of-bias assessment was performed.
Data synthesis	12	Describe the methods used for summarizing, handling, synthesizing, tabulating, visualizing, and analyzing the bibliometric data.	Section 2.4, pages 6–8 (data harmonization, VOSviewer/CiteSpace settings, threshold sensitivity analyses, fractional counting, R/bibliometrix analyses, complete-year treatment, CPD, citations per year, and JCR IF data); Supplementary Table S2 (standardization and thesaurus rules).
**Results**			
Descriptive findings/statistics	13	Provide details of the search and selection process and report descriptive bibliometric findings, including records retrieved and included.	Section 2.3, pages 5–6, and Figure 1, page 8 (records retrieved, screened, overlapping, and included); Section 3.1, pages 8–14 (annual output and country/institution distributions).
Schematic map and trend	14	Present maps and trends using appropriate bibliometric software and indicators.	Figures 1–9 and Sections 3.1–3.5, pages 8–27 (screening/overlap, publication trends, collaboration networks, source-journal analyses, dual-map overlay, co-citation networks, keyword clusters, and trending topics).
Tabulation and summarizing the findings	15	Summarize and organize findings in appropriate tables, figures, and subsections relevant to the main topic.	Sections 3.1–3.5, pages 8–27; Tables 1–8 and Figures 1–9; Supplementary Tables S4–S5 provide the extended highly cited publication rankings.
Synthesis of findings	16	Synthesize the findings, identify research gaps, and propose future directions where applicable.	Discussion, Sections 4.1–4.3, pages 28–33, and Section 5, Conclusions, pages 34–35 (synthesis of growth, collaboration, knowledge base, implementation settings, outcome gaps, and future priorities).
**Discussion**			
Summary of evidence	17	Summarize the main findings in general and accessible terms.	Section 4.1, Principal Findings, page 28 (summary of the main bibliometric findings across WoSCC and Scopus).
Interpretation	18	Interpret the findings consistently with the results, including explanations for observed trends, patterns, similarities, and differences.	Sections 4.1–4.3, pages 28–33 (interpretation of geographic distribution, collaboration, evidence evolution, withdrawal management, primary care, pharmacist involvement, long-term care, patient acceptability, nonpharmacological support, safety outcomes, and implementation implications).
Strengths and limitations	19	Discuss the strengths and limitations of the bibliometric review.	Dual-database design and rationale: Introduction, pages 4–5, and Section 2.1, page 5. Limitations: Section 4.4, pages 33–34 (database/language coverage, bibliometric scope, and database-specific analyses).

Section/Topic	Item No.	Checklist item	Reported location in the manuscript
Conclusion(s)	20	Provide a general interpretation of the results with respect to the review objectives and potential implications.	Section 5, Conclusions, pages 34–35 (field evolution, patient-centered medication-safety framework, stakeholder-oriented implementation implications, digital approaches, and future research priorities).

Table S4. Publications ranked 1–20 by total citations in WoSCC.

Rank	Paper	Title	DOI	Total citations	TC per year
1	TANNENBAUM C, 2014, JAMA INTERN MED	Reduction of Inappropriate Benzodiazepine Prescriptions Among Older Adults Through Direct Patient Education: The EMPOWER Cluster Randomized Trial.	10.1001/jamainternmed.2014.949	495	38.08
2	LADER M, 2011, ADDICTION	Benzodiazepines revisited—will we ever learn?	10.1111/j.1360-0443.2011.03563.x	412	25.75
3	CLEGG A, 2011, AGE AGEING	Which medications to avoid in people at risk of delirium: a systematic review	10.1093/ageing/afq140	382	23.88
4	IYER S, 2008, DRUG AGING	Medication withdrawal trials in people aged 65 years and older: a systematic review	10.2165/0002512-200825120-00004	269	14.16
5	LADER M, 2014, BRIT J CLIN PHARMACO	Benzodiazepine harm: how can it be reduced?	10.1111/j.1365-2125.2012.04418.x	233	17.92
6	POTTIE K, 2018, CAN FAM PHYSICIAN	Deprescribing benzodiazepine receptor agonists: Evidence-based clinical practice guideline	NA	229	25.44
7	LADER M, 2009, CNS DRUGS	Withdrawing benzodiazepines in primary care	10.2165/0023210-200923010-00002	185	10.28
8	REEVE E, 2017, EUR J CLIN PHARMACOL	A systematic review of interventions to deprescribe benzodiazepines and other hypnotics among older people	10.1007/s00228-017-2257-8	143	14.30
9	HOEL RW, 2021, MAYO CLIN PROC	Polypharmacy management in older patients	10.1016/j.mayocp.2020.06.012	142	23.67
10	FARRELL B, 2015, PLOS ONE	What are priorities for deprescribing for elderly patients? Capturing the voice of practitioners: a modified delphi process	10.1371/journal.pone.0122246	134	11.17
11	FANG SY, 2009, DRUG ALCOHOL DEPEN	Predictors of the incidence and discontinuation of long-term use of benzodiazepines: A population-based study	10.1016/j.drugalcdep.2009.04.017	102	5.67
12	GOULD RL, 2014, BRIT J PSYCHIAT	Interventions for reducing benzodiazepine use in older people: meta-analysis of randomised controlled trials	10.1192/bjp.bp.113.126003	100	7.69
13	SIRDIFIELD C, 2013, BMC FAM PRACT	General practitioners' experiences and perceptions of benzodiazepine prescribing: systematic review and meta-synthesis	10.1186/1471-2296-14-191	99	7.07
14	NISHTALA PS, 2009, DRUG AGING	Impact of Residential Medication Management Reviews on Drug Burden Index in Aged-Care Homes: A Retrospective Analysis	10.2165/11316440-000000000-00000	98	5.44
15	REEVE E, 2013, INT J CLIN PHARM-NET	Development and validation of the patients' attitudes towards deprescribing (PATD) questionnaire	10.1007/s11096-012-9704-5	93	6.64

Rank	Paper	Title	DOI	Total citations	TC per year
16	SLUGGETT JK, 2017, PHARMACOL RES	Medication management policy, practice and research in Australian residential aged care: Current and future directions	10.1016/j.phrs.2016.12.011	92	9.20
17	MARTIN P, 2017, BMJ OPEN	A realist evaluation of patients' decisions to deprescribe in the EMPOWER trial	10.1136/bmjopen-2017-015959	89	8.90
18	DILLS H, 2018, J AM MED DIR ASSOC	Deprescribing Medications for Chronic Diseases Management in Primary Care Settings: A Systematic Review of Randomized Controlled Trials	10.1016/j.jamda.2018.06.021	89	9.89
19	LEMOINE P, 2012, EXPERT OPIN PHARMACO	Prolonged-release formulation of melatonin (Circadin) for the treatment of insomnia	10.1517/14656566.2012.667076	88	5.87
20	BRODATY H, 2018, J AM MED DIR ASSOC	Antipsychotic Deprescription for Older Adults in Long-term Care: The HALT Study	10.1016/j.jamda.2018.05.002	86	9.56

TC, total citations. TC per year was calculated using publication age through 2026. Citation counts are database-specific.

Table S5. Publications ranked 1–20 by total citations in Scopus.

Rank	Paper	Title	DOI	Total citations	TC per year
1	TANNENBAUM C, 2014, JAMA INTERN MED	Reduction of inappropriate benzodiazepine prescriptions among older adults through direct patient education: the EMPOWER cluster randomized trial	10.1001/jamainternmed.2014.949	531	40.85
2	CLEGG A, 2011, AGE AGEING	Which medications to avoid in people at risk of delirium: a systematic review	10.1093/ageing/afq140	480	30.00
3	MARTIN P, 2018, JAMA	Effect of a pharmacist-led educational intervention on inappropriate medication prescriptions in older adults: the D-PRESCRIBE randomized clinical trial	10.1001/jama.2018.16131	325	36.11
4	IYER S, 2008, DRUGS AGING	Medication withdrawal trials in people aged 65 years and older: a systematic review	10.2165/0002512-200825120-00004	319	16.79
5	LADER M, 2014, BR J CLIN PHARMACOL	Benzodiazepine harm: how can it be reduced?	10.1111/j.1365-2125.2012.04418.x	261	20.08
6	POTTIE K, 2018, CAN FAM PHYS	Deprescribing benzodiazepine receptor agonists: Evidence-based clinical practice guideline	NA	260	28.89
7	MARKOTA M, 2016, MAYO CLIN PROC	Benzodiazepine Use in Older Adults: Dangers, Management, and Alternative Therapies	10.1016/j.mayocp.2016.07.024	231	21.00
8	LADER M, 2009, CNS DRUGS	Withdrawing Benzodiazepines in Primary Care	10.2165/0023210-200923010-00002	226	12.56
9	BRETT J, 2015, AUST PRESCR	Management of benzodiazepine misuse and dependence	10.18773/austprescr.2015.055	206	17.17
10	REEVE E, 2017, EUR J CLIN PHARMACOL	A systematic review of interventions to deprescribe benzodiazepines and other hypnotics among older people	10.1007/s00228-017-2257-8	157	15.70
11	HOEL RW, 2021, MAYO CLIN PROC	Polypharmacy Management in Older Patients	10.1016/j.mayocp.2020.06.012	155	25.83
12	FARRELL B, 2015, PLOS ONE	What are priorities for deprescribing for elderly patients? Capturing the voice of practitioners: A modified Delphi process	10.1371/journal.pone.0122246	146	12.17
13	GOULD RL, 2014, BR J PSYCHIATRY	Interventions for reducing benzodiazepine use in older people: Meta-analysis of randomised controlled trials	10.1192/bjp.bp.113.126003	117	9.00
14	LEE J, 2021, BMJ OPEN	Deprescribing fall-risk increasing drugs (FRIDs) for the prevention of falls and fall-related complications: a systematic review and meta-analysis	10.1136/bmjopen-2019-035978	112	18.67
15	NISHTALA PS, 2009, DRUGS AGING	Impact of residential medication management reviews on drug burden index in aged-care homes: A retrospective analysis	10.2165/11316440-000000000-00000	108	6.00
16	FANG SY, 2009, DRUG ALCOHOL DEPEND	Predictors of the incidence and discontinuation of long-term use of benzodiazepines: A population-based study	10.1016/j.drugalcdep.2009.04.017	108	6.00
17	SCOTT IA, 2013, EVID BASED MED	Deciding when to stop: Towards evidence-based deprescribing of drugs in older populations	10.1136/eb-2012-100930	105	7.50
18	WHITMAN A, 2018, SUPPORT CARE CANCER	Pharmacist-led medication assessment and deprescribing intervention for older adults with cancer and polypharmacy: a pilot study	10.1007/s00520-018-4281-3	103	11.44
19	WESTBURY JL, 2018, MED J AUST	RedUSe: Reducing antipsychotic and benzodiazepine prescribing in residential aged care facilities	10.5694/mja17.00857	94	10.44
20	BRODATY H, 2018, J AM MED DIR ASSOC	Antipsychotic Deprescription for Older Adults in Long-term Care: The HALT Study	10.1016/j.jamda.2018.05.002	91	10.11

TC, total citations. TC per year was calculated using publication age through 2026. Citation counts are database-specific.
